# *Arabidopsis* cell wall composition determines disease resistance specificity and fitness

**DOI:** 10.1073/pnas.2010243118

**Published:** 2021-01-28

**Authors:** Antonio Molina, Eva Miedes, Laura Bacete, Tinguaro Rodríguez, Hugo Mélida, Nicolas Denancé, Andrea Sánchez-Vallet, Marie-Pierre Rivière, Gemma López, Amandine Freydier, Xavier Barlet, Sivakumar Pattathil, Michael Hahn, Deborah Goffner

**Affiliations:** ^a^Centro de Biotecnología y Genómica de Plantas, Universidad Politécnica de Madrid (UPM)–Instituto Nacional de Investigación y Tecnología Agraria y Alimentaria (INIA), 28223 Pozuelo de Alarcón, Madrid, Spain;; ^b^Departamento de Biotecnología-Biología Vegetal, Escuela Técnica Superior de Ingeniería Agronómica, Alimentaria y de Biosistemas, Universidad Politécnica de Madrid (UPM), 28040 Madrid, Spain;; ^c^Department of Statistics and Operations Research, Faculty of Mathematics, Complutense University of Madrid, 28040 Madrid, Spain;; ^d^Interdisciplinary Mathematics Institute, Complutense University of Madrid, 28040 Madrid, Spain;; ^e^Laboratoire de Recherche en Sciences Végétales, Université Toulouse III-Paul Sabatier, Centre National de la Recherche Scientifique, 31326 Castanet-Tolosan Cedex, France;; ^f^Laboratory of Plant-Microbe Interactions, Université Toulouse III-Paul Sabatier, Institut National de Recherche pour l’Agriculture, l’Alimentation et l’Environnement, Centre National de la Recherche Scientifique, 31326 Castanet‐Tolosan Cedex, France;; ^g^Complex Carbohydrate Research Center, University of Georgia, Athens, GA 30602-4712

**Keywords:** cell wall, disease resistance, immunity, fitness, glycomics

## Abstract

Plant cell walls are complex structures subject to dynamic remodeling in response to developmental and environmental cues and play essential functions in disease resistance responses. We tested the specific contribution of plant cell walls to immunity by determining the susceptibility of a set of *Arabidopsis* cell wall mutants (*cwm*) to pathogens with different parasitic styles: a vascular bacterium, a necrotrophic fungus, and a biotrophic oomycete. Remarkably, most *cwm* mutants tested (29/34; 85.3%) showed alterations in their resistance responses to at least one of these pathogens in comparison to wild-type plants, illustrating the relevance of wall composition in determining disease-resistance phenotypes. We found that the enhanced resistance of *cwm* plants to the necrotrophic and vascular pathogens negatively impacted *cwm* fitness traits, such as biomass and seed yield. Enhanced resistance of *cwm* plants is not only mediated by canonical immune pathways, like those modulated by phytohormones or microbe-associated molecular patterns, which are not deregulated in the *cwm* tested. Pectin-enriched wall fractions isolated from *cwm* plants triggered immune responses in wild-type plants, suggesting that wall-mediated defensive pathways might contribute to *cwm* resistance. Cell walls of *cwm* plants show a high diversity of composition alterations as revealed by glycome profiling that detect specific wall carbohydrate moieties. Mathematical analysis of glycome profiling data identified correlations between the amounts of specific wall carbohydrate moieties and disease resistance phenotypes of *cwm* plants. These data support the relevant and specific function of plant wall composition in plant immune response modulation and in balancing disease resistance/development trade-offs.

## Significance

Plant cells are surrounded by an extracellular matrix known as the cell wall. We have analyzed the contribution of the *Arabidopsis* cell wall to disease resistance to pathogens with different parasitic styles. Here, we demonstrate that plant cell walls are determinants of immune responses since modification of their composition in a set of *Arabidopsis* cell wall mutants has an impact on their disease resistance and fitness phenotypes. In these genotypes, we identified specific correlations between the amounts of specific wall carbohydrate epitopes and disease resistance/fitness phenotypes through mathematical analyses. These data support the relevant and specific function of plant cell wall composition in plant immune responses and provide the basis for using wall traits in crop breeding programs.

Plants are under continuous pathogen threats that might compromise their survival and reproduction. To cope with these threats, plants have evolved a plethora of resistance mechanisms, which are either constitutively expressed or induced after pathogen attack (1–4). One common resistance mechanism to all plant cells is the presence of a cell wall that shields plants from pathogen invasion. The cell wall acts first as a passive barrier that pathogens have to hydrolyze by secreting cell wall–degrading enzymes for infection progression but also functions as a reservoir of antimicrobial compounds (5–7). Plant cell walls are also a source of carbohydrate moieties that are released during wall degradation and could act as damage-associated molecular patterns (DAMPs) triggering plant immune responses upon their perception by plant pattern recognition receptors (PRRs) (6–12). Plant walls are complex and dynamic structures that consist of a primary wall composed of carbohydrate-based polymers—cellulose, pectic polysaccharides (homogalacturonan, rhamnogalacturonan (RGI), and RGII), hemicelluloses (xyloglucan and xylans) and minor polysaccharides—and of structural glycoproteins (13). In addition, to reinforce their structure, some plant cells deposit a secondary wall that is mainly composed of cellulose, hemicelluloses (mostly xylans), and lignin (14, 15). The biosynthesis, transport, deposition, remodeling, and turnover of cell walls, along with the regulation of these processes, involve ∼10% of genes encoded in plants genomes (16, 17).

Modifications of cell wall composition and structure occur during plant development but also upon plant exposure to environmental stresses (e.g., drought or pathogen attack) or treatments with chemicals disrupting wall biosynthesis (e.g., isoxaben). These wall modifications have a direct effect on cell wall integrity (CWI) and can initiate molecular adaptive mechanisms, such as cell wall composition remodeling and defensive responses activation (12, 18–21). CWI alteration also occurs in plants impaired in or overexpressing cell wall–related genes. Some of these plants/mutants show altered disease-resistance phenotypes that were initially associated with the misadaptation of pathogens to overcome the modified wall structures of these genotypes (5, 7, 22–27). However, activation of defensive pathways takes place in the majority of these mutants/overexpressing lines with wall alterations (5, 7, 22–27). For instance, impairment of cellulose synthesis for secondary cell walls by inactivating cellulose synthase subunits, as it occurs in *Arabidopsis thaliana* irregular xylem mutants (*irx1*, *irx3*, and *irx5*), or for primary cell walls, as it occurs in *Arabidopsis *iso*x*aben–resistant (*ixr1*), also known as constitutive expression of**vegetative storage protein 1 (*cev1*) mutant, results in constitutive activation of some canonical defensive responses and enhanced resistance to different pathogens. For example, *irx1-6* shows enhanced resistance to the necrotrophic fungus *Plectosphaerella cucumerina* (*Pc*) and the vascular bacterium *Ralstonia pseudosolanacearum* (*Rp*) (formerly *Ralstonia solanacearum* (28–31)). Similarly, alteration of the biosynthesis and/or structure of wall pectins (e.g., degree of methyl-esterification) can also affect pathogen resistance (8, 32–37). Moreover, modification of glucuronoxylans and xyloglucans structure also has impacts on disease resistance, as it occurs in the *Arabidopsis de-etiolated 3* (*det3*) mutant that shows enhanced resistance to *Pc* (38, 39) or in *agb1* mutant (impaired in Gβ subunit of the heterotrimeric G protein), which has reduced xylose content and shows enhanced susceptibility to several pathogens, including *Pc*, the biotrophic oomycete *Hyaloperonospora arabidopsidis* (*Hpa*), and the hemibiotrophic bacterium *Pseudomonas syringae* (40–43). Also, modification of the degree of acetylation of wall polysaccharides and of lignin composition affect disease resistance, growth, and adaptation to environmental changes of plants (12, 44–46). Interestingly, cell wall modification can also result in contrasting disease resistance effects as illustrated by the *arr6-3* mutant that shows enhanced resistance to *Pc* but is highly susceptible to *Rp* (20).

Alteration of CWI can initiate the release of DAMPs that regulate plant immune responses in a similar way to those triggered by microbe-associated molecular patterns (MAMPs) (6, 24, 47). Despite the diversity of glycan structures of plant cell walls, only a limited number of wall-associated DAMPs have been identified so far, including some oligosaccharides structures derived from β-1,3-glucan (callose), cellulose, xyloglucan, mannan, homogalacturonan, and arabinoxylan polysaccharides of plant cell walls (6, 8–10, 12, 47–51). Modification of CWI also leads to developmental phenotype alterations (e.g., reduced plant size and biomass or fertility), indicating that the cell wall contributes to plant fitness (5, 19, 52, 53). Notwithstanding the evidence of the roles of plant cell walls in immunity and fitness, correlations between variations in cell wall carbohydrate moiety composition and specific phenotypes have not been described until recently (12, 41, 54).

We have investigated the specific contribution of plant cell wall to disease resistance by testing the susceptibility to three different pathogens of a large set of *Arabidopsis* cell wall mutants (*cwm*; *n* = 34). We found that a significant proportion of these mutants (29 of 34; 85.3%) showed altered disease-resistance phenotypes, supporting a more relevant function of such extracellular layer in plant immunity than currently considered. Here, we demonstrate, combining mathematical analyses and glycome profiling, that the content of specific wall glycan moieties in *cwm* plants correlates with some of their disease resistance and fitness phenotypes, providing a link between plant cell wall composition and plant development/immunity phenotypes.

## Results

### *Arabidopsis* Cell Wall Composition Specifically Contributes to Disease- Resistance Responses.

To determine the specific function of plant cell wall in immunity, we selected two large sets of *Arabidopsis cwm* and tested their resistance to three pathogens with different parasitic styles: the necrotrophic fungus *Pc*, the vascular bacterium *Rp*, and the biotrophic oomycete *Hpa*. The first set of *cwm* included 18 previously described mutants, such as irregular xylem (*irx1-6*, *irx2-1*, *irx3-1*, *irx6-1*, *irx8-1*, *irx10-1*, and *irx12-1*), powdery mildew resistance (*pmr5-1* and *pmr6-1*) mutants, and *det3-1*, *fra3-1*, *wat1-1*, *arr6-3*, *agb1-1*, *exp1-1*, *araf1-1*, *araf2-1*, and *ctl2-1* lines (*SI Appendix*, Figs. S1 and S2). The second *cwm* set was composed of 16 transfer DNA (T-DNA) insertional mutants, which were impaired in orthologs of *Zinnia elegans* genes differentially expressed during xylogenesis, a process involving secondary wall biosynthesis (*SI Appendix*, Fig. S1) (22). The majority of these mutations (e.g., those in T-DNA insertional mutants) resulted in loss-of-function mutants, as expression of the impaired genes was not detected by RT-PCR or led to truncated proteins, but hypomorphic alleles were also included in the analyses (*SI Appendix*, Figs. S1 and S2). We checked the resistance phenotypes of these 34 *cwm* lines and their wild-type counterparts (Col-0, Ws, or La-*er*) upon infection with *Pc*, *Rp*, or *Hpa* either by evaluating plants macroscopic disease symptoms caused by *Rp* and *Pc* and assigning Disease Rating values (DR) or by determining *Hpa* sporangiophores formation on plant leaves and conidiospore production by these sporangiophores per plant fresh weight. In all these disease-resistance analyses, susceptible and resistant control genotypes (mainly for Col-0 background) were included for comparison as follows: 1) *irx1-6* as control of resistance (*cr*) for *Pc* and *Rp,* and La*-er* and Col-0 wild-type ecotypes as *cr* (gene for gene resistance) for *Hpa* (Col-0 and La-*er*/Ws, mutant backgrounds, respectively); 2) controls of susceptibility (*cs*) for Col-0 genotypes were *agb1-1* for *Pc*, *arr6-3* for *Rp*, and *NahG* plants, defective in salicylic acid (SA) pathway, for *Hpa*; and 3) *cs* of *Hpa* for Ws and La-*er* genotypes were *eds1-1* (Ws) and *eds1-2* (Col-0) alleles, respectively, which are impaired in the gene for gene resistance (20, 29, 31, 39, 55‒56). In addition, *irx6-1* (Ws) and *irx3-1* (La-*er*) were included as *cr* of *Pc* and *Pc/Rp *for Ws and La*-er*, respectively (20).

**Fig. 1.**
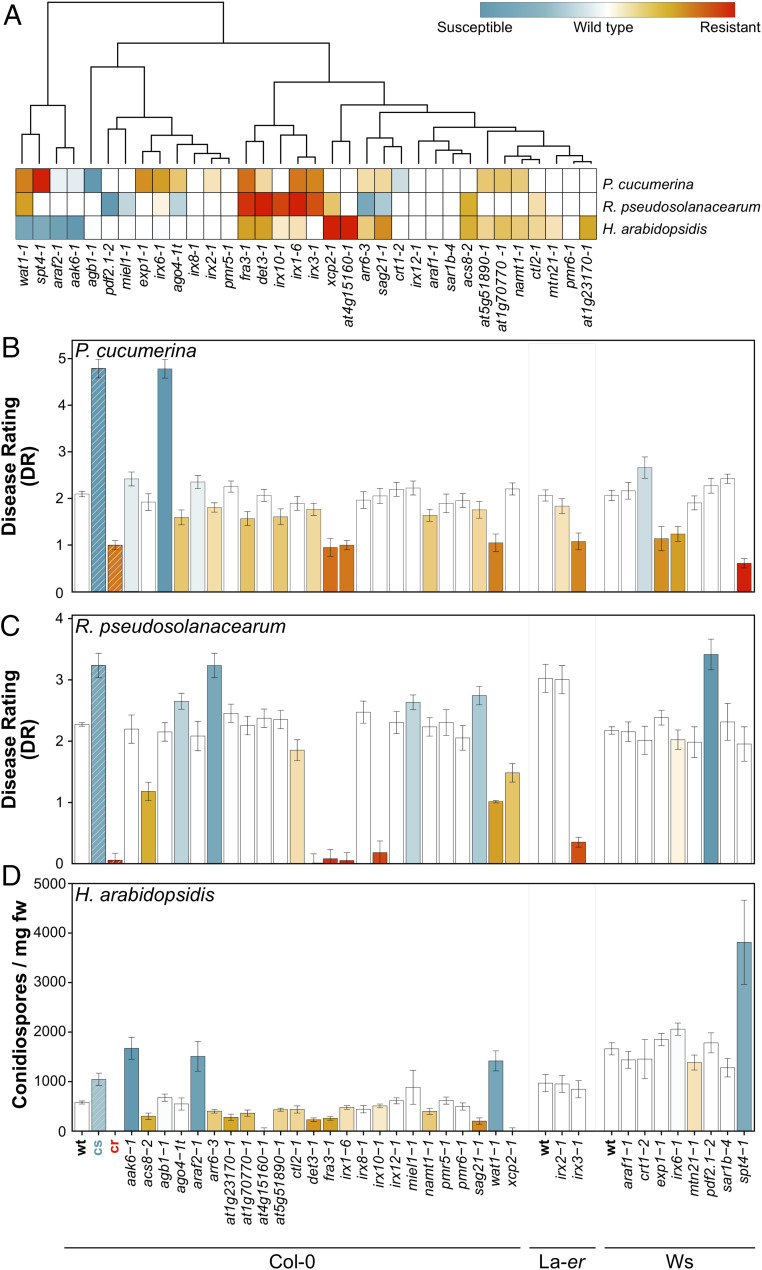
*Arabidopsis* cell wall mutants show alterations of their disease-resistance phenotypes in comparison to wild-type plants. (*A*) Clustering of disease-resistance phenotypes of *Arabidopsis* cell wall mutants to *P. cucumerina* (*Pc*), *R.* *pseudosolanacearum *(*Rp*), and *H. arabidopsidis* (*Hpa*). Clusters were computed using Euclidean distances using disease-resistance indexes relative to wild-type (wt) plants (DR for *Pc* and *Rp*; number of conidiospores per milligram of rosette fresh weight (mg fw) for *Hpa*). The color-coding of the corresponding columns/squares indicates the level of the resistance phenotype, from susceptible (blue) to resistant (red), that have been established for each pathogen tested. Colored squares/columns indicate values of significant differences compared with wt values (ANOVA nonbalanced analysis and Dunnett’s test, *P* ≤ 0.05). (*B*) DR (average ± SD) of cell wall mutants and wt plants (Col-0, La-*er*, and Ws backgrounds; *n* > 10) at 7 dpi with the necrotrophic fungus *Pc*. DR varies from 0 (noninfected plants) to 5 (dead plants). The *irx1-6* and *agb1-1* mutants were included as *cr* and *cs*, respectively. (*C*) DR (average ± SD) of wt and mutants (*n* > 10) at 8 dpi with bacterium *Rp*. DR varies between 0 (no symptoms) and 4 (dead plants). *irx1-6* and *arr6-3* mutants were included as *cr* and *cs*, respectively. (*D*) Number of conidiospore/milligram fresh weight in wt and mutant plants (average ± SD; *n* > 20) at 7 dpi with the oomycete *Hpa.* La*-er* and Col-0 wild-type ecotypes were included as *cr* for Col-0 and La-*er*/Ws mutant backgrounds, respectively, and *NahG* plants (Col-0), *eds1-1* (Ws), and *eds1-2* (Col-0) alleles were used as *cs* for Col-0, Ws, and La-*er* mutant backgrounds, respectively (*SI Appendix*, Fig. S3). Data in *B*–*D* are from one representative experiment of the three performed that gave similar results. References and details of *cwm* mutants are listed in *SI Appendix*, Figs. S1 and S2, and the DR and conidiospore/mg fw values (average ± SD) are shown in *SI Appendix*, Fig. S3.

We found that 29 of the 34 *cwm* lines tested (85.3%) showed, in comparison to wild-type plants, altered resistance responses (mainly enhanced resistance) to at least one of these pathogens: 20 of 34 mutants to *Pc* (58.8%), 19 of 34 to *Hpa* (55.9%), and 15 of 34 mutants to *Rp* (44.2%; Fig. 1 and *SI Appendix*, Fig. S3). Cluster analyses of these phenotypes identified some specific groups of *cwm* mutants with similar disease-resistance phenotypes but also a high diversity of disease-resistance phenotypes illustrated by mutants with unique phenotypes (Fig. 1*A*). Remarkably, 15 of the 34 mutants showed enhanced resistance to more than 1 pathogen and 3 mutants to all 3 (*fra3-1* and *det3-1* and the previously characterized *irx1-6*) (29, 31) (Fig. 1 and *SI Appendix*, Fig. S3). For *Pc*, several mutants (e.g., *det3-1*, *fra3-1*, *at1g70770-1*, *ago4-1t*, *sag21-1*, *irx2-1*, *at5g51890*, or *arr6-3*) showed lower DR than wild-type plants and enhanced resistance, whereas several mutants showed higher DR than wild-type plants and enhanced susceptibility (e.g., *xcp2-1*, *crt1-1*, *araf2-1*, and *akk6-2*), but the levels of disease resistance values were weaker than those of *cr* (*irx1-6/irx3-1/irx6-1*) and *cs* (*agb1-1*) controls, respectively (Fig. 1*B* and *SI Appendix*, Fig. S3) (29, 40). *Pc* resistance phenotypes were further validated by infection of a representative set of additional alleles of some mutants (*SI Appendix*, Fig. S4). In the analysis of resistance to *Rs*, we identified 4 mutants (*pdf2.1-2*, *ago4-t*, *sag21-1*, and *miel1-1*) that showed, like *arr6-3*, enhanced susceptibility and more severe disease symptoms and DR than wild-type plants and 10 mutants showing lower DR and enhanced resistance (*det3-1*, *xcp2-1*, *irx10-1*, *fra3-1*, *wat1-1*, *ctl2-1*, *irx1-6*, *acs8-2*, *irx6-1*, and *irx3-1*) than their corresponding wild-type plants (Fig. 1*C* and *SI Appendix*, Fig. S3). Except for *fra3-1* and *irx10-1*, the enhanced resistance of these 10 mutants was weaker than that of the previously characterized *irx1-6*, *irx3-1*, or *wat1-1* partially resistant genotypes, whereas only *pdf2.1-2* plants were as susceptible as the recently described hypersusceptible *arr6-3* plants (20, 29, 57; Fig. 1*C* and *SI Appendix*, Fig. S3). Notably, we found 14 mutants with enhanced resistance to the biotroph *Hpa* (e.g., *det3-1*, *xcp2-1*, *at1g23170-1*, *fra3-1*, *at1g70770-1*, *acs8-2*, *at4g15160-1*, *namt1-1*, *sag21-1*, *at5g518-1*, *ctl1-1*, *irx1-6*, *at5g51890-1*, and *arr6-3*), showing two of them (*at4g15160-1* and *xcp2-1*) a reduction in conidiospore production similar to that of *cr* control, whereas four lines (*wat1-1*, *at3g47510-1*, *irx6-1*, and *spt4-1*) were more susceptible than wild-type plants to this oomycete, but their susceptibility was weaker than that of *NahG* or *agb1-1* (Col-0), *eds1-1* (Ws), or *eds1-2* (La-*er*) included as *cs* (Fig. 1*D* and *SI Appendix*, Fig. S3). All these susceptible genotypes included as controls developed a significantly higher number of sporangiophores in their leaves/cotyledons than the corresponding wild-type plants, the *cr* genotypes, or *cwm* lines showing enhanced resistance (*SI Appendix*, Fig. S5), further supporting their disease-resistance phenotype classification based on conidiospore production. These data pointed to a relevant function of the cell wall composition in resistance to different types of pathogens.

### Enhanced Resistance of *cwm* Plants to *Pc* and *Rp* Negatively Impacts Plant Fitness.

**Fig. 2.**
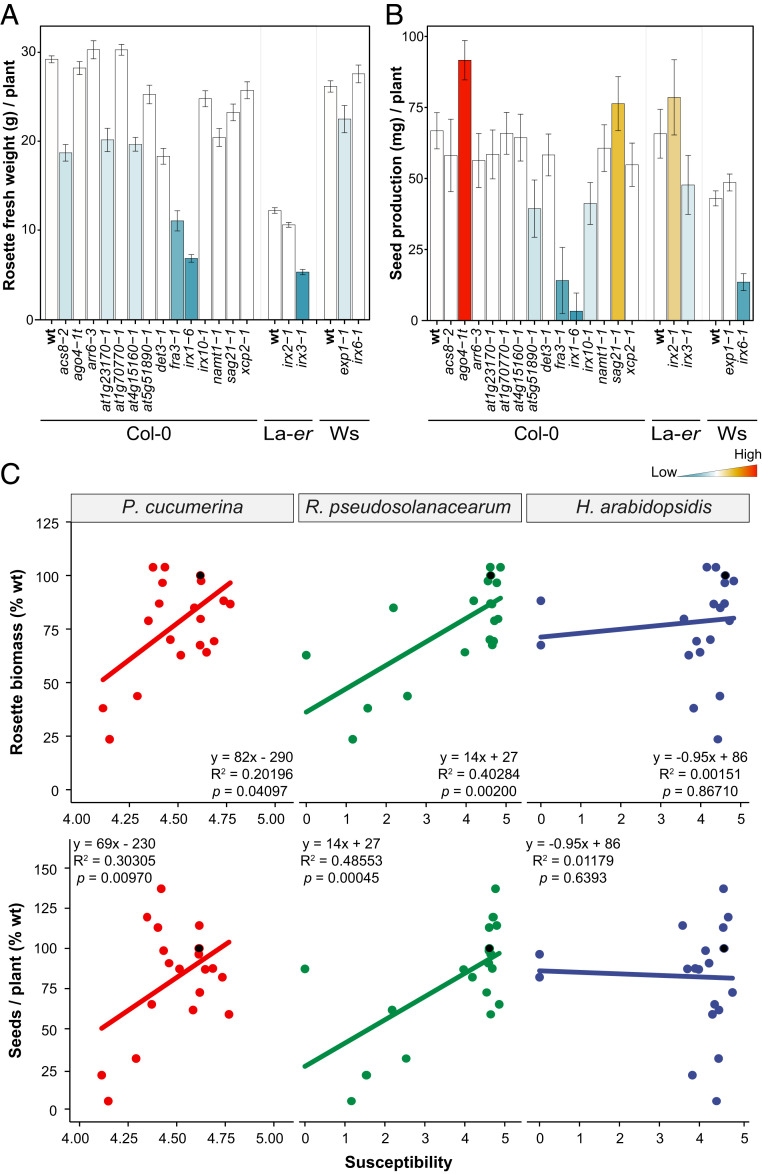
*Arabidopsis* cell wall mutants show associated resistance/fitness trade-offs. (*A*) Rosette fresh weight biomass (average g/plant ± SD) of 4-wk-old mutants and wild-type (wt) plants (Col-0, La-*er*, and Ws backgrounds). (*B*) Seed yield (average milligram/plant ± SD) of wt plants and mutants at the end of reproductive cycle. Data are the average of 10 plants. The column color indicates significant differences compared with wt values (ANOVA nonbalanced analysis and Dunnett’s test, *P* ≤ 0.05), with higher and lower values than wt indicated in red and blue, respectively. This is one representative experiment of the three performed that gave similar results. (*C*) Correlation analysis between biotic stress susceptibility to pathogens (*Pc*, *Rp*, and *Hpa*) and fitness parameters (seed yield and rosette biomass) of 18 *cwm* mutants and wt plants (Col-0, La-*er*, and Ws backgrounds). The average response information of each genotype (dot in the graph) is expressed in relation to that of the reference wt plant (black dot; value of 100% at the *y*-axes). Disease resistance susceptibility ratios were log-transformed, and accordingly, *x*-axes range from 0 (lower susceptibility) to 5 (greater susceptibility), with the wt plants situated at 4.72 = *ln* (1 + 100). A linear model was fitted for each combination and correlations determined. Fitted equations, *R*-squares, and *P* values are indicated in the insets of the graphs. The *x*-axes of the figures involving *Pc* are enlarged in the 4 to 5 range for better visualization.

The overall developmental phenotypes (e.g., rosette size and leaf architecture) of the majority of *cwm* tested did not differ significantly from those of wild-type plants. This is in contrast with the previously described dwarf/altered phenotypes of *irx1-6*, *irx3-1*, *fra3-1*, or *det3-1* mutants and that found here for *at5g51890-1* (*SI Appendix*, Fig. S6 and references in *SI Appendix*, Fig. S1). Since disease resistance/developmental growth trade-offs have been described in *Arabidopsis* (4, 58, 59), we selected 18 *cwm* mutants from representative clusters of resistance phenotypes (Fig. 1*A*) and different ecotype backgrounds, and we measured vegetative (rosette biomass) and reproductive (seed production) traits related to fitness under growth conditions with no limitation of nutrients and water and no infection. Rosette biomass (fresh weight) of 4-wk-old plants was, in comparison to wild-type plants, reduced (between 30 and 80%) in 9 out of 18 *cwm* tested (*det3-1*, *irx1-6*, *at4g15160-1*, *acs8-2*, *namt1-1*, *at1g23170-1*, *fra3-1*, *irx6-1*, and *irx3-1*), and no significant increase in rosette biomass was observed in any of *cwm* lines (Fig. 2*A*). Seed production at the end of the reproductive cycle was significantly reduced, in comparison to wild-type plants, in six mutants (*irx10-1*, *irx1-6*, *at5g51890-1*, *fra3-1*, *irx3-1*, and *irx6-1*) and notably increased in two *cwm* lines (*ago4-t1* and *sag21-1)* (Fig. 2*B*). Both fitness traits were negatively affected only in three mutants, *irx1-6*, *fra3-1*, and *irx3-1*, as described previously (29, 53), suggesting that these two traits are decoupled.

We next determined in this subset of 18 *cwm* mutants if their fitness alteration was associated with their resistance/susceptibility phenotypes. Correlation analyses were performed after conversion of DR and fitness data to percentage susceptibility ratios (with respect to each ecotype’s wild-type value), followed by least-squares (LS) means estimation. A negative correlation was found in *cwm* plants between both rosette biomass and seed production and resistance to *Pc* (*P* = 0.04097 and *R*^2^ = 020196 for seed yield and *P* = 0.0097 and *R*^2^ = 0.30305 for biomass) and *Rp* (*P* = 0.002 and *R*^2^ = 0.40284 for seeds yield and *P* = 0.00045 and *R*^2^ = 0.48553 for biomass) (Fig. 2*C*). In contrast, a negative association was not found between the resistance phenotype to *Hpa* of *cwm* plants and their seeds yield (*P* = 0.8671 and *R*^2^ = 0.00151) or biomass (*P* = 0.6393 and *R*^2^ = 0.01179) (Fig. 2*C*). These results indicated that trade-offs between resistance to *Pc*/*Rp* and plant development exist. Of note, we did not find, among the *cwm* mutants with enhanced resistance, any with higher seed yield or rosette biomass than wild-type plants (Fig. 2*C*), indicating that *cwm* defensive responses associated to CWI alteration are costly for plant development.

Associations between resistance to *Pc* or *Rp* and tolerance to abiotic stresses (e.g., drought, desiccation, and salinity) have been reported (29, 54). Accordingly, we quantified the tolerance to desiccation (survival percentage rate upon rewatering after desiccation) of the 18 *cwm* genotypes. We found that six of them (*det3-1*, *irx1-6*, *fra3-1*, *irx3-1*, *irx10-1*, and *irx2-1*) were more tolerant to desiccation than the wild-type plants (*SI Appendix*, Fig. S7*A*). Of note, a positive correlation was found between desiccation tolerance of *cwm* plants and disease resistance to either *Pc* (*P* = 0.03141 and *R*^2^ = 0.22128) or *Rp* (*P* = 7.196 × 10^−6^ and *R*^2^ = 0.66227) but not to *Hpa* (*P* = 0.5254 and *R*^2^ = 0.02155; *SI Appendix*, Fig. S7*B*). These results are in line with previous findings indicating that resistance to *Pc*/*Rp* and desiccation tolerance could be linked traits (29, 57).

### Enhanced Resistance Phenotypes of *cwm* Plants Are Associated with Different Alterations of Their Cell Wall Compositions.

**Fig. 3.**
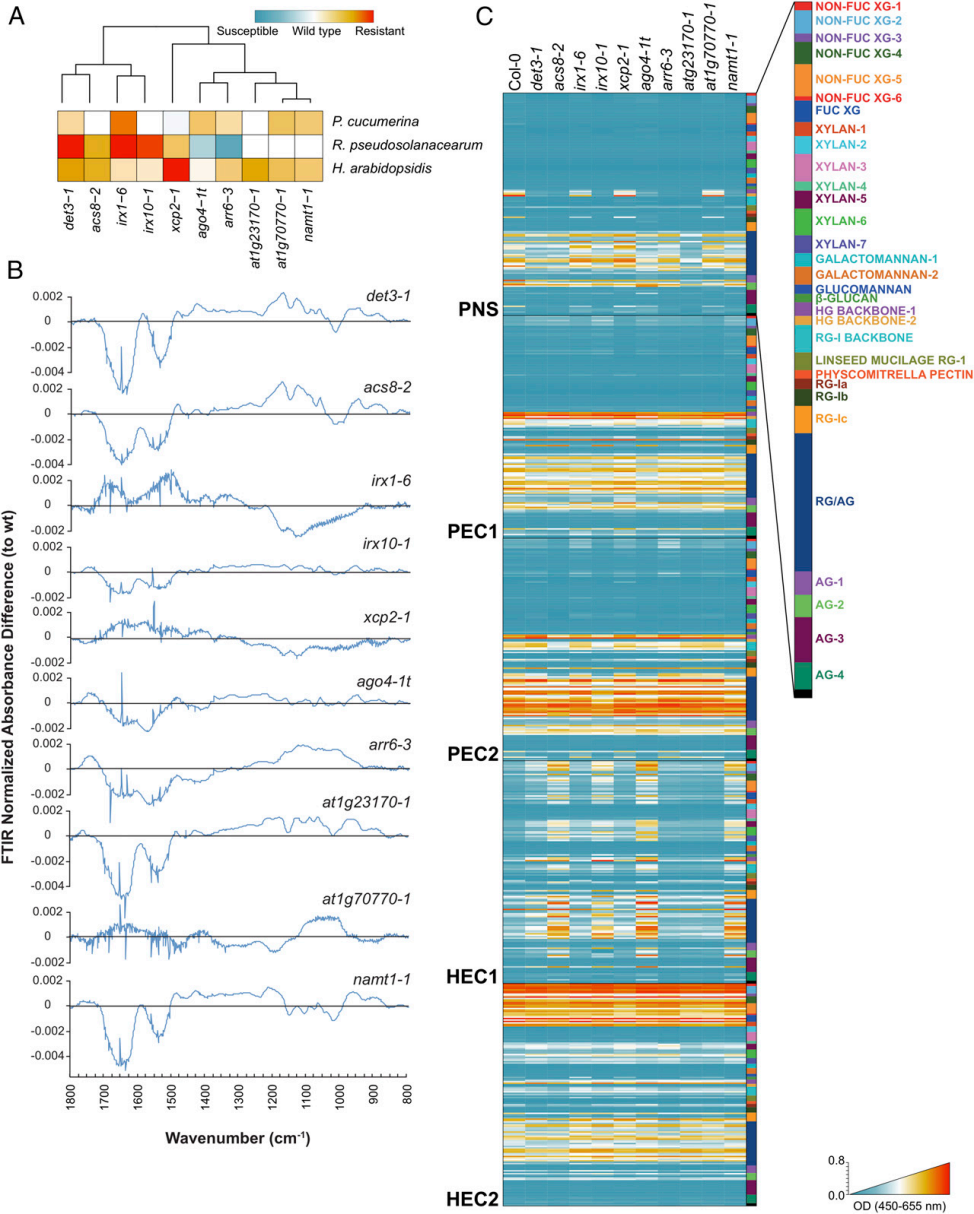
Cell wall analyses by FTIR spectroscopy and glycome profiling of a core set of *Arabidopsis* cell wall mutants. (*A*) Selection of a core set of representative mutants with different levels of disease resistance to *Pc*, *Rp*, and *Hpa*. Clusters were computed by Euclidean distances using disease-resistance indexes relative to wild-type (wt) plants. (*B*) Cell wall FTIR difference spectra of mutants and wt plants (Col-0). The black line indicates wt values, and values over this line are differential FTIR spectra in the mutants tested. (*C*) Heatmaps of glycome profiling of cell wall extracts (PNS, PEC1, PEC2, HEC1, and HEC2) of *cwm* and wt (Col-0) rosette leaves of 25-d-old plants (see Dataset S1 for details). Heatmaps depict antibody binding strength based on optical density (OD) indicated as a color gradient ranging from blue (no binding) to red (strongest binding). The list of monoclonal antibodies used for glycome profiling of each fraction and wall structures recognized by them are indicated (*Right*) (see Dataset S1 for details). Data represent average values of two independent experiments (*n* > 10).

We next determined the putative correlations between the observed resistance phenotypes of *cwm* plants and their wall composition (e.g., cellulose, neutral sugars, and uronic acid content). Of the subset of 18 mutants used in trade-off analyses, only 9 have been previously characterized as cell wall mutants (*SI Appendix*, Fig. S1), whereas 9 were putative wall mutants (22). We found, in comparison to wild-type plants, differences in the composition of the walls of the majority of this subset of 18 mutants; *at1g23170-1*, *at1g70770-1*, *xcp2-1 ago4-1t*, and *irx6-1* had reduced and *acs8-2* had increased levels of cellulose; *det3-1* and *irx1-6* possessed less pectic uronic acids; and noncrystalline neutral sugars levels were increased in *arr6-3* and decreased in *at1g70770* and *acs8-2* (*SI Appendix*, Fig. S8). These data confirmed that the majority of nine putative *cwm* initially selected showed wall alterations. Since these biochemical characterizations of the cell wall composition of *cwm* plants were not very precise, we narrowed down the collection to a set of 10 mutants, representing six different clusters with different resistance phenotypes, and performed a deeper cell wall profiling (Fig. 3*A*). We subjected mutants and wild-type purified cell walls to Fourier-Transform InfraRed (FTIR) spectroscopy that can assign wall polymers and functional groups to different wavenumbers of the FTIR spectra (60). Differential FTIR spectra obtained after digital subtraction of the wild-type values from the mutants showed clearly that *xcp2-1*, *namt2-1*, *acs8-2*, *at1g70770-1*, *at1g23170-1*, and *ago4-1t* were cell wall mutants with biochemical alterations that differ from those observed in the previously characterized *det3-1*, *irx1-6*, *irx10-1*, and *arr6-3* wall mutants (Fig. 3*B*; 21, 28). Since some of the wavenumbers of the differential FTIR spectra were associated to lignin components (wavenumbers at 1,515, 1,630, and 1,720 cm^−1^), we determined total lignin content, and we found that it was altered in four mutants (*det3-1*, *irx1-6*, *namt1-1*, and *at1g23170-1*), further supporting that these genotypes were mainly affected in secondary wall composition (*SI Appendix*, Fig. S9).

**Fig. 4.**
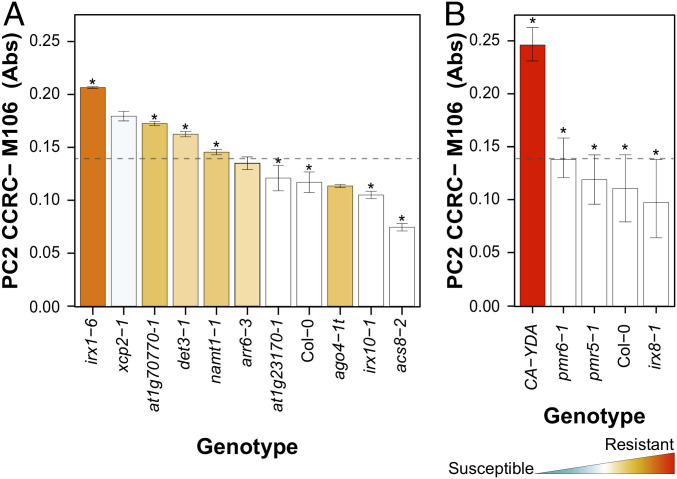
CRT analyses correlate wall composition and disease-resistance phenotypes of the *Arabidopsis* cell wall mutants. (*A*) Biological validation of CRT model for resistance to *Pc* with cell wall mutants from six different clusters (Fig. 3*A*). The absolute value (average ± SD) of the epitope signal detected by CCR-M106 antibody is shown. Columns are colored according to the resistance level of the corresponding mutant, from red (resistant) to blue (susceptible), in comparison with wild-type (wt) level of resistance (white column; ANOVA nonbalanced analysis, Dunnett’s test, *P* ≤ 0.05). The scale used is the same of that in Figs. 1 and 3*A*. The absorbance cutoff value for considering a mutant as resistant or susceptible/wt phenotype, as determined by CRT, is indicated by the dotted lines. The mutant genotypes which follow the CRT model are marked with an asterisk. (*B*) Biological validation of the CRT model with *pmr5-1*, *pmr6-1*, *and irx8-1* mutants that do not show enhanced resistance to *Pc* and *CA-YDA* plants that show enhanced resistance to the fungus. All these mutants follow the absorbance cutoff value predicted by the CRT model, and, accordingly, they are marked with an asterisk.

These classical cell wall analyses were complemented with an in-depth characterization of *cwm* wall composition by glycome profiling using a collection of 155 glycan-directed antibodies, recognizing diverse cell wall substructures (61, 62). These analyses were carried out on the following five sequential wall extracts obtained from rosettes of each of plant genotypes: protein and neutral sugars (PNS), two pectin (PEC1 and PEC2), and two hemicellulose (HEC1 and HEC2) wall extracts, which are known to be enriched in different glycans (61, 62). Glycome profiling confirmed that all of the selected mutants showed significant differences in the abundances of some wall glycan epitopes in comparison to wild-type plants (Fig. 3*C* and Dataset S1) and also corroborated the diversity of wall compositions in the selected wall mutants. The relative abundances of some specific wall epitopes in the PEC1 and PEC2 glycome profiles (e.g., fucosylated-xyloglucan) showed opposite patterns in the resistant *cwm* mutants in comparison to those showing wild-type or hypersusceptible phenotypes, suggesting some kind of correlation between specific wall epitopes and disease resistance (Dataset S1). To test this hypothesis, we performed a model analysis to uncover and generalize the potential relationships between each mutant’s glycomic data (that act as independent or explanatory variables) and disease resistance to *Pc*, *Rp*, or *Hpa* as response variables. We used a nonparametric Classification and Regression Tree (CRT) methodology for these analyses, which provides simple and interpretable classification models with almost no statistical assumptions (*SI Appendix*, Fig. S10*A* and *Materials and Methods*). CRT identified a set of antibodies whose reaction values explained, with an estimated cross-validation accuracy between 83.43% and 84.34% (in 10 *cwm* and wild-type genotypes), the resistance phenotypes of *cwm* plants (*SI Appendix*, Fig. S10*B* and Table S1). For example, the abundance of fucosylated-xyloglucans (recognized by CCRC-M106 antibody) correlated with the level of resistance to *Pc* and explained the response phenotypes of 8 out of 11 genotypes tested (Fig. 4*A*). Similarly, CCR5-M5 (detecting a yet undefined RGI epitope) correlated with the resistance to *Rp* (8 out of 10 *cwm* genotypes), and CCRC-M174 (detecting galactomannan) and CCRC-M106 (detecting fucosylated-xyloglucans) explained the resistance to *Hpa* (8 out of 10 *cwm* genotypes) (*SI Appendix*, Figs. S10*B* and S11). Additional carbohydrate moieties may also contribute to explain a mutant’s disease-resistance phenotypes but with lower accuracy values (*SI Appendix*, Table S1). To further validate the association of fucosylated-xyloglucan (CCRC-M106) with the *Pc* disease-resistance phenotype, we performed glycomic analyses with selected antibodies on three additional mutants (*pmr5-1*, *pmr6-1*, and *irx8-1*), with disease resistance to *Pc* similar to that of wild-type plants (Col-0; Fig. 1*A*), and on *CA-YDA* plants that overexpress the constitutive active YODA MAP3K and show enhanced resistance to *Pc* and additional pathogens (63). As predicted by the model, walls of *CA-YDA* plants, but not those of *pmr5-1*, *pmr6-1*, and *irx8-1*, showed an enhanced accumulation of the fucosylated-xyloglucan epitope recognized by CCRC-M106 in comparison to wild-type plant cell walls (Fig. 4*B*).

Similar CRT analyses were then performed with the fitness parameters (biomass and seed yield, acting as dependent variables) of these 10 *cwm* mutants and wild-type plants. Of note, we found a relationship between the reaction signal of some antibodies recognizing some particular carbohydrate moieties and these fitness traits, which explained between 87.31% and 87.62% of the phenotypes; CCRC-M22 (selective for a six-linked β-galactan epitope in RGI and arabinogalactan) explained biomass phenotypes, and the levels of epitope detected by CCRC-M175 (galactomannans) and CCRC-M170 (acetylated mannans) correlated with seed yield (*SI Appendix*, Figs. S10*B* and S12 and Table S1). Similarly, we found a relationship between tolerance to desiccation and the epitope recognized by the JIM101 antibody (detecting an RGI epitope) that explained 83.16% of the phenotypes (*SI Appendix*, Table S1). Together, these data suggest that the cell wall composition of *Arabidopsis* is a determinant of plant developmental phenotypes and resistance/tolerance to biotic and abiotic stresses.

### Disease-Resistance Responses of Cell Wall Mutants Is Not Associated with the Differential Regulation of Canonical Defensive Pathways.

The molecular defensive mechanisms underlying the enhanced resistance/susceptibility of the *cwm* lines were further investigated by qRT-PCR determination of the expression of defense genes in noninfected and *Pc*-inoculated plants (1-d post inoculation [dpi]). The tested genes are either up-regulated by MAMPs (e.g., *WRKY33*, *PHI1*, *CYP81F2*, and *PAD3*), CWI alteration (*At1g51890*; 64), or defensive phytohormones (*PR1*, *LOX3*, *PR4*, *LTP3*, and *PDF1.2*: gene markers of SA, ethylene [ET], jasmonic acid [JA], abscisic acid, and ET plus JA, respectively). We clustered the expression levels of these genes in *cwm* with their resistance phenotypes to identify potential correlations, and only a significant cluster between expression of *LTP3* in noninoculated *irx1-6* plants and disease resistance to *Pc* was found (*SI Appendix*, Fig. S13*A*), as previously described (29). In *Pc*-inoculated plants, only one cluster was found associated to *PR1* expression, but it did not explain the *cwm* resistance phenotypes to any pathogen, since it includes two mutants and wild-type plants (*SI Appendix*, Fig. S13*B*). These data indicated that a constitutive expression or enhanced up-regulation upon infection of phytohormone-, or MAMP-triggered- or CWI-related genes might not explain the *cwm*-enhanced resistance or susceptibility phenotypes observed.

We have recently shown that pectin wall fractions (PEC1 and PEC2) of Col-0 and cell wall mutant *arr6-3* (Fig. 1*C*) contain potential glycan-derived DAMPs that regulate immune responses when applied to Col-0 wild-type plants (20). Further biochemical subfractionation and characterization of *arr6-3* PEC1 has led to the identification of an arabinoxylan pentasaccharide (3^3^-α-l-arabinofuranosyl-xylotetraose) as a novel active DAMP, triggering immune responses such as Ca^+2^ burst and mitogen-activated protein kinases (MPKs) phosphorylation in Col-0 wild-type plants (51). Similar immune responses triggered by elicitor activities have been described in wall extracts of additional *Arabidopsis* wall mutants (12, 44). Given these previous data, we investigated and found that the PEC1 and PEC2 wall extracts from *cwm* plants, like those of *arr6-3* included as control, triggered early immune responses, such as Ca^+2^ bursts, upon their application to *Arabidopsis* Col-0^AEQ^ lines expressing the apoaequorin Ca^+2^ sensor protein (*35S::Apoaequorin*_cyt_) (20, 47; *SI Appendix*, Fig. S14). To further determine whether the signaling mechanisms regulating *cwm* PEC1-mediated Ca^+2^ bursts were similar to those triggered by other MAMPs/DAMPs, we generated an *agb1-2*^AEQ^ line and tested Ca^+2^ bursts upon PEC1 treatment, as *agb1-2* is impaired in immune responses triggered by several MAMPs such as flg22, elf18, and chitin (65, 66). Col-0^AEQ^ and *agb1-2* ^AEQ^ lines treated either with PEC1 from Col-0 or the most active *cwm* fractions (*SI Appendix*, Fig. S14) showed similar Ca^+2^ bursts, which contrast with the reduced Ca^+2^ burst of *agb1-2*^AEQ^ line treated with flg22 in comparison to Col-0^AEQ^ (*SI Appendix*, Fig. S15*A*), indicating that PEC1-triggered immunity does not require the immune regulator AGB1. To further characterize PEC1-mediated immunity, we tested the activity of Col-0 and *cwm* PEC-1 in triggering MPK phosphorylation in Col-0 and in *bak1-5* mutant that is impaired in BAK1 coreceptor required for several immune responses (67). We found that PEC1 from *cwm* genotypes trigger MPK phosphorylation, which was, in general, higher than that of PEC1 from Col-0, and we observed that MPK phosphorylation was not impaired in PEC1-treated *bak1-5* plants, which was different from the significant reduction in MPK phosphorylation observed in *bak1-5* treated with flg22 when compared with Col-0–treated plants (*SI Appendix*, Fig. S15*B*). These data suggest that these wall extracts might contain additional DAMPs or increased amounts of DAMPs, in comparison to wild-type wall extracts that might regulate *Arabidopsis* immune responses through signaling pathways that do not seem to involve the immune regulator AGB1 and the BAK1 coreceptor. The immune activity of these cell wall fractions (e.g., PEC1) might contribute to and explain the disease-resistance phenotypes of the *Arabidopsis* cell wall mutants tested.

## Discussion

Plant cell walls are important components of both preexisting and inducible plant defense mechanisms against pathogen infection (5, 6, 18, 19). Accordingly, modifications of cell wall composition and structure in some mutants or transgenic lines have been demonstrated to result in the alteration of their resistance phenotypes to different pathogens, including hemibiotrophic (e.g., *P. syringae*; 8, 29, 68) and vascular (e.g., *Rp*; 29, 40, 57) bacteria and necrotrophic (e.g., *Pc* and *Botrytis cinerea*; 21, 29–32, 36, 39, 42, 57, 69) or biotrophic fungi (e.g., *Erysiphe* sp. 28; 70–74). For example, 15 of the wall mutants analyzed in this study have been previously described to show differential disease resistance phenotypes to one or two pathogens (e.g., *irx1-6* and *irx3-1*), and in a few cases, like *irx1-6*, *agb1-1*, and *arr6-3* used as controls in the analyses, their resistance to three or more pathogens have been determined (Fig. 1 and *SI Appendix*, Fig. S1). Despite these previous data, specific correlations between wall composition/structure and the resistance phenotypes and/or immune responses of these plant genotypes have not been described.

Here, we have determined the contribution of *Arabidopsis* cell walls to disease-resistance responses against three pathogens with very different parasitic styles by selecting a large set of cell wall mutants that includes well-characterized and putative wall mutants (22). Given the different molecular bases of *Arabidopsis* resistance to the three pathogens tested (*Pc*, *Rp*, and *Hpa*; 20, 29, 39, 40, 57, 75) and the putative diversity of cell wall alterations in the mutants screened, we initially anticipated that we could obtain a global view of *Arabidopsis* cell wall contribution to resistance. Notably, we found that 85.3% of the cell wall mutants tested (29 of 34) showed, in comparison to wild-type plants, differential phenotypes (enhanced resistance, mainly, or susceptibility in a few cases) to at least one of the three pathogens tested. Of note, we have identified different clusters containing one or several mutants with specific phenotypes (e.g., from enhanced resistance to the three pathogens to specific resistance to one pathogen) (Fig. 1*A*). These data support the diverse and significant functions of cell walls on plant disease resistance responses to vascular and necrotrophic pathogens, as previously described (20, 29, 57). Our data also identified a contribution of cell walls to plant resistance to biotrophic oomycetes, such as *Hpa* (Fig. 1*C* and *SI Appendix*, Figs. S3 and S5), which is in line with the described wall function in *Arabidopsis* resistance to biotrophic fungi causing powdery mildew diseases (70–73, 75). The proportion of mutants with differential disease resistance phenotypes identified in this screening is several orders of magnitude higher than the expected proportion that would be obtained in blind, unbiased disease-resistance screenings using T-DNA or chemically mutagenized plant populations. It can be anticipated that a similar proportion of genotypes to that obtained here would be found if a biased screening was performed with *Arabidopsis* mutants impaired in known components of key defensive pathways, such as phytohormone signaling or MAMP-triggered immunity (76, 77). Therefore, the data obtained here with the set of *cwm* plants strongly supports the relevant function of cell walls in plant immunity and disease resistance to different pathogens.

The genetic and molecular basis of *Arabidopsis* resistance to the three pathogens analyzed here differ significantly: 1) plant resistance to *Hpa* mainly depends on activation of effector-triggered immunity (ETI) and of the SA pathway (see resistance and susceptible controls in Fig. 1*D* and *SI Appendix*, Figs. S3 and S5); 2) disease resistance to *Rp* is mediated by ET, and just a few examples of ETI responses have been described; and 3) *Arabidopsis* resistance to necrotrophic fungi, including *Pc*, has been shown to depend on hormones signaling (mainly ET and JA, but also SA) and on the synthesis of tryptophan-derived metabolites (such as camalexin and indole glucosinolates), and few examples of ETI-mediated resistance have been described so far (78, 79). This lack of source of resistance genes triggering ETI to control *Rp* and necrotrophic fungi, such as *Pc* or *B. cinerea*, might explain the strong incidence of the diseases caused by these two types of pathogens in crops and the associated yield losses since breeding programs have not been effective in selecting traits conferring enhanced resistant to these pathogens (80, 81). This contrasts with the effectiveness of breeding programs in controlling biotrophic pathogens, such as oomycetes (e.g., *Hpa*) causing downy mildews or fungi (e.g., *Erysiphe* sp.) causing powdery mildew diseases (79, 82). Our data indicate that CWI disruption could be, initially, an effective strategy in the control of diseases caused by necrotrophic and vascular pathogens and, therefore, that a genomic-assisted breeding selection of CWI-associated traits could be used in breeding programs, as suggested previously for other crop traits, such as biomass digestibility (83). However, modification of cell wall composition and structure usually results in alterations of plant developmental phenotypes (e.g., reduced plant size, biomass, or fertility) that impact fitness (19, 84). In line with these previous data, we describe here a negative correlation between fitness parameters, such as rosette biomass and seed production, of the cell wall mutants tested and their enhanced resistance to vascular and necrotrophic pathogens (e.g., *Rp* and *Pc*) (Fig. 2*C*). These trade-offs associated to increased resistance to these pathogens are also probably hampering the selection of crop traits conferring improved resistance. In contrast, we have not found in the genotypes tested associated trade-offs to the enhanced resistance to the biotroph *Hpa* (Fig. 2*C*), indicating that some wall-associated traits identified here might be of interest for improving resistance to biotrophic pathogens.

Plant cell walls (primary and secondary) are complex and dynamic structures composed mainly of carbohydrate-based polymers of differing monosaccharide and glycosyl-linkage compositions (13). Among the genotypes included in our analysis, we selected 18 previously described wall mutants showing a great diversity of wall alterations, such as reduction/alteration of the content/decorations of cellulose (*irx1-6*, *irx3-1*, *irx6-1*, *irx2-1*, or *ctl2*), xylan (*irx10-1*), glucuronoxylan (*irx8-1)*, pectin (*pmr6-1*, *pmr5-1*, *arr6-3*, and *irx8-1*), xyloglucans (*agb1-1*), or lignin (*irx12*-*1*) (see *SI Appendix*, Fig. S1 for references), or impairment in glycan transport or *in muro* biosynthesis of wall components (e.g., *ctrl1-1*, *det3-1*, *wat1-1*, and *fra3-1)* (see *SI Appendix*, Fig. S1 for references). We also tested 16 putative cell wall mutants (*SI Appendix*, Figs. S1 and S3), including some that have been recently characterized as wall mutants (e.g., *araf2-1* and *araf1-1* impaired in arabinan-containing pectins or *xcp2-1* and *arr6-3*), and seven mutants whose wall alterations have been demonstrated for the first time here (Fig. 3, *SI Appendix*, Figs. S8 and S9, and Dataset S1). These data corroborate that the majority of genotypes initially selected are bona fide *Arabidopsis* wall mutants. Cell wall modifications identified by FTIR spectroscopy or biochemical analyses in the mutants from the six phenotypic clusters selected (Fig. 3*A* and *SI Appendix*, Figs. S8 and S9) were not precise enough to find specific associations between wall composition and disease-resistance phenotypes. Chemically extracted cell wall fractions (e.g., PEC1 and PEC2) contain mixtures of carbohydrate moieties derived from various polymer classes and can be enriched in certain carbohydrates detectable by glycome profiling. We show here that glycome profiling analysis of these extracts provides a more precise picture of wall modifications impacting disease resistance.

Our data show that mathematical modeling by CRT of glycome profiling of plant genotypes provides detailed and biologically consistent links between cell wall composition and disease resistance/fitness phenotypes, as it has been previously reported for the determination of cell wall digestibility of plant genotype biomass (12, 85, 86). The CRT algorithm used here allows for both the identification of variables (cell wall components recognized by some antibodies) and the definition of cut-points on these variables, separating mathematically in different branches and nodes the genotypes belonging to different phenotypic classes (best, equal, or worse than wild-type phenotypes). Since CRT is based on binary branching, it obtains more pure or homogenous nodes (in terms of their class composition) in contrast to other supervised classification methods (e.g., linear discriminant analysis, logistic regression, random forest, or classification trees). Using CRT, we have identified significant epitope associations explaining as much as 84.34% of the disease-resistance phenotypes tested (Fig. 4*B* and *SI Appendix*, Fig. S11*B* and Table S1). Remarkably, the abundance of fucosylated-xyloglucan (detected by CCRC-M106), RGIa (CCRC-M5), and galactomannan (CCRC-M174)/fucosylated-xyloglucan (CCRC-M106) in the cell walls of the mutants correlated with the level of resistance to *Pc*, *Rp*, and *Hpa*, respectively (Fig. 4 *A* and *B* and *SI Appendix*, Figs. S11 and S12). The relevance of xyloglucan and xylose content in *Arabidopsis* disease resistance to *Pc* has been previously described (39) and is further validated here by the content of wall epitope recognized by the CCRC-M106 antibody, which is enhanced in the *Pc*-resistant *CA-YDA* plants (Fig. 4*B*; 60). In contrast, galactomannan and RGIa/fucosylated-xyloglucan contribution to *Rp* and *Hpa* resistance, respectively, have not been previously described. Notably, we have also demonstrated here that the level of a six-linked β-galactan epitope present in RGI/arabinogalactans (CCRC-M22) and of acetylated-mannan/galactomannan (CCRC-M170/CCRC-M175) correlated with rosette biomass and seed production, respectively, indicating that wall composition can also determine plant fitness (*SI Appendix*, Fig. S13). These data are in accordance with previous results showing that high-density quantitative glycan microarrays, used in conjunction with association mapping, can detect pertinent variations related to plant cell wall genetics (12, 85, 86). Since the specificity of the carbohydrate moieties, recognized by some of the antibodies identified here, has not been fully established yet, in contrast to other antibodies of the glycomics collection (62), it cannot be excluded that disease resistance/fitness traits could be associated with other types of wall epitopes than those described here.

Various hypotheses have been put forward to explain why modification of cell wall composition often appears to enhance rather than reduce plant disease resistance (7, 12, 87). These hypotheses include strategies to avoid plant wall breakdown by microbial cell wall–degrading enzymes either by reshuffling (“masking”) wall composition or by releasing inhibitor proteins targeting microbial enzymes, but also the activation of immune responses upon recognition by PRRs of released elicitor-active molecules (DAMPs) from incorrectly assembled plant cell walls (20, 51). Our data support that some particular immune pathways are differentially regulated in some of the mutants, but their expression patterns do not explain their disease-resistance phenotypes (*SI Appendix*, Fig. S13). Notably, we also show that cell wall fractions of some *cwm* plants trigger immune responses, suggesting that they might contain additional DAMPs or enhanced levels of DAMPs in comparison to wild-type fractions (*SI Appendix*, Fig. S14), as it has been described recently to occur in other *Arabidopsis* wall mutants (12, 20, 44, 51). In this regard, our approach pointed to a role of fucosylated-xyloglucans and galactomannans in plant disease resistance, and, interestingly, recent reports have proposed these β-1, 4-linked components of hemicelluloses as potential plant DAMPs (49, 88). Although these reports do not allow us to narrow down the type of xyloglucans and galactomannans that our methodology has found to be involved in immunity, the fact that such structures can trigger defense responses in plants is at least promising. Of note, immune responses activated by the *cwm* wall fractions, such as PEC1, do not seem to require key regulators of canonical immune responses mediated by MAMPs, such as AGB1 and BAK1 (*SI Appendix*, Fig. S15), suggesting that novel signaling mechanisms and molecular components might be involved in the activation of immune responses activated by plant cell walls glycans. A growing number of plant cell wall–associated DAMPs have been identified so far; however, the mechanisms involved in their perception by plant PRRs are poorly characterized. Notably, several of these plant cell wall DAMPs trigger enhanced disease resistance responses when applied exogenously to *Arabidopsis* and crops (49, 51, 88). In line with these previous data, DAMP-triggered immunity, together with the canonical immune pathways that might be constitutively expressed or primed for stronger activation upon pathogen infection in some of the cell wall mutants analyzed, would contribute to regulating their immune responses and disease-resistance phenotypes. The characterization of these *cwm* defensive responses, and the wall DAMPs and plant PRRs involved in their activation, deserves further attention to understand these novel wall-associated immune responses.

## Materials and Methods

### Plant Materials and Growth Conditions.

*Arabidopsis* genotypes used in this study and oligonucleotides used for T-DNA insertional mutant characterization are listed in *SI Appendix*, Fig. S1 and Table S2. For plants used in *Rp* assays, seeds were germinated on Murashige and Skoog (MS) medium and then grown in Jiffy pots (www.jiffygroup.com) in a chamber at 22 °C, with a 9-h light period and a light intensity of 200 μmol ⋅ m^−2^ ⋅ sec^−1^ and 50% relative humidity. Plants used in *Pc* and *Hpa* disease resistance and fitness experiments were grown on soil in a growth chamber as described (20). Plant rosette biomass was determined on 4-wk-old plants (*n* = 10), and seeds were harvested at 8 wk after plants (*n* = 10) completed their vegetative cycle. Experiments were repeated at least three times with similar results. Genotyping of T-DNA insertional mutants was performed by PCR amplification of DNA extracted from mutant leaves following established protocols (20) and the oligonucleotides indicated in *SI Appendix*, Table S2. The Simple Sequence Length Polymorphisms (SSLP) markers nga119 or nga151 were used to confirm Ws background of the mutant tested.

### Pathogen Growth Conditions and Plant Infections.

*Pc* Brigitte Mauch-Mani (BMM) strain and *Hpa* (isolates Noco2, Emwa1, and Cala) were grown as described (20). *Rp* (strains GMI1000 and RD15) were grown at 28 °C on Bacto-Agar (15 mg/mL) and glucose (5 mg/mL) medium. For *Pc* infection, 3-wk-old plants (*n* > 10) were sprayed with a suspension spore (4 × 10^6^ spores/mL) of virulent *Pc* BMM isolate, progression of the infection was followed by visual evaluation of DR at different dpi, and the average DR, from 0 to 5, was scored as follows: 0 = no symptoms, 1 = plant with some necrotic spots, 2 = one or two necrotic leaves, 3 = three or more leaves showing necrosis, 4 = more than half of the plant showing profuse necrosis, and 5 = decayed/dead plant (39). For *Hpa* assays, 12-d-old plants (*n* > 20) were sprayed with a conidiospore suspension (2 × 10^4^ spores/mL) of virulent isolates (Noco2, Emwa1, and Cala for plants in Col-0, Ws, and La-*er* backgrounds, respectively). Then, plants were incubated under short day conditions (10-h illumination) for 7 d, and the aerial parts of all plants were harvested and shaken in water, released conidiospores counted, and the average per milligram plant fresh weight determined (47). For *Rp* infections, roots of 4-wk-old plants (*n* > 10) were dipped into a bacterial suspension (5 × 10^7^ cfu/mL) of virulent strains GMI1000 (for Col-0 and La-*er*) or RD15 (for Ws). Following inoculation, plants were transferred to a growth chamber under the following conditions: 12 h photoperiod, 27 °C, and 80% relative humidity. The average DR was scored in leaves as follows: 0 = no symptoms, 1 = 25% wilted leaves, 2 = 50% wilted leaves, 3 = 75% wilted leaves, and 4 = 100% wilted leaves (dead plant; 74). All pathogen resistance assays were repeated at least three times, and in all these experiments, susceptible and resistant control genotypes were included for comparisons (Fig. 1 and *SI Appendix*, Figs. S3 and S4).

### Plant Cell Wall Purification, Fractionation, and Analyses.

Cell wall alcohol insoluble residues (AIR) were prepared from 25-d-old *Arabidopsis* plants according to ref. 89, and noncellulosic fraction, uronic acid, and crystalline cellulose and lignin contents were determined as previously described (63, 90). FTIR spectroscopy determination was done with discs prepared from mixtures of purified AIR and KBr (1:100, w:w) using a Graseby-Specac press. FTIR spectra were recorded and analyzed as described (91). Lignin-like material was quantified by the Klason gravimetric method with minor modifications (92). AIR fractions were subjected to sequential chemical extraction with increasingly harsh reagents in order to isolate fractions enriched in various cell wall components as previously described: PNS fraction, pectic fractions (PEC1 and PEC2), and hemicellulosic fractions (HEC1 and HEC2) (20, 89). Glycome profiling of the cell wall fractions was carried out by enzyme-linked immunosorbent assay (ELISA) using a toolkit of plant cell wall–directed monoclonal antibodies as previously described (see *SI Appendix*; 61, 62). Monoclonal antibodies are annotated in the database at glycomics.ccrc.uga.edu/wall2/antibodies/antibodyHome.html, and specific links to the antibodies are included in Dataset S1.

### Gene Expression Analyses.

Total RNA was extracted using the RNeasy Mini Kit (Qiagen) from *Arabidopsis* wild-type plants and mutants (*SI Appendix*, Fig. S2) and from mock-treated or *Pc* BMM–inoculated and rosettes (*n* > 25) at 1 dpi (four biological replicates), as reported previously (39). Quantitative real-time PCR amplification or RT-PCR detection were carried out as previously described (47). Oligonucleotides used for gene expression are detailed on *SI Appendix*, Table S3. The expression levels of each gene, relative to *UBC21* (*AT5G25760*) expression, were determined using the Pfaffl method (93).

### Clustering and Statistical Analyses.

Heatmaps and cluster aggrupation (Figs. 1*A* and 3*A* and *SI Appendix*, Fig. S13) were calculated using “ggplots” R package version 3.0.3. Clusters in Figs. 1*A* and 3*A* were computed using Euclidean distances using disease resistance indexes relative to wild-type plants (DR for *Pc* and *Rp*; the number of conidiospores per milligram of rosette fresh weight for *Hpa*). Clusters in *SI Appendix*, Fig. S13 were computed using Euclidean distances for absolute gene expression levels and disease indexes.

ANOVA models were fitted for each of the response variable (resistance to *Pc*, *Rp*, *Ha*, biomass and seed yield, and desiccation tolerance) (Figs. 1 and 2 *A* and *B* and *SI Appendix*, Fig. S7*A*) and each ecotype (Col-0, Ws, or La-*er*). LS means of these models were then obtained, providing a single estimation of the average response level (e.g., mean DR for both *Pc* and *Rp*, conidiospores/milligram plant fresh weight for *Ha*, seed yield in milligram and rosette fresh weight in milligram, and survival rate after desiccation) for each genotype. Afterward, correlation analyses (*SI Appendix*, Fig. S16) between biotic resistance and fitness features/desiccation were obtained by determining the ratio of each genotype LS mean to that of the corresponding wild-type ecotype for each response variable (e.g., percentage susceptibility levels with respect to wild-type plants). A logarithmic model was fitted for each combination of the biotic susceptibility ratios with the fitness and abiotic susceptibility ratios to analyze their correlations (see Fig. 2*C* and *SI Appendix*, Fig. S7*B* for the fitted equations, *R*-squares, and *P* values). For more details, see *SI Appendix*, *Supplementary Material and Methods*.

CRT predictive classification model (*SI Appendix*, Fig. S11), correlating wall composition with disease resistance and fitness phenotypes, was done by performing, first, a paired comparison analysis to assign *Arabidopsis* wild-type and *cwm* mutant genotypes into a class (e.g., a categorical valuation), which represents its status compared to wild-type plants with a similar performance (class equal), significantly better, or significantly worse than wild-type ones. The CRT method was then applied to link this class status to glycomics data. To avoid overfitting the data, the tree growing process of each CRT model was limited to a single binary branching to select a single antibody and its optimal cutoff point. The actual predictive capability or accuracy of the resulting classification tree models is evaluated as the percentage of correctly classified genotypes obtained through a 10-fold cross-validation process, replicated 100 times. The correlation and paired comparison analyses were implemented using the SAS software (*glm* and *corr* procedures), while the CRT classification model fitting and validation were implemented using Python (*scikit-learn* library: Data Set 2_CRTPythonscript, or see link: https://github.com/tinguarorg/PNAS_CellWall.git). See *SI Appendix*, *Supplementary Material and Methods* for further details.

## Supplementary Material

Supplementary File

Supplementary File

Supplementary File

## Data Availability

All study data are included in the article and/or supporting information.
