# Supplementary material for: Arabidopsis cell wall composition determines disease resistance specificity and fitness
Source: Proc Natl Acad Sci U S A. 2021 Jan 28;118(5):e2010243118. doi: 10.1073/pnas.2010243118 (PMC7865177; doi:10.1073/pnas.2010243118)
Supplement: Supplementary File [file pnas.2010243118.sapp.pdf]

PNAS
www.pnas.org

Supplementary Information for

Arabidopsis cell wall composition determines disease resistance specificity and fitness

Antonio Molina, Eva Miedes, Laura Bacete, Tinguaro Rodríguez, Hugo Mélida, Nicolas Denancé, Andrea Sánchez-Vallet, Marie-Pierre Rivière, Gemma López, Amandine Freydier, Xavier Barlet, Sivakumar Pattathil, Michael Hahn and Deborah Goffner

Correspondence to Antonio Molina
Email: antonio.molina@upm.es

This PDF file includes:

Supplementary text
Figures S1 to S16
Tables S1 to S3
SI References 1-46

Other supplementary materials for this manuscript include the following:

Dataset S1 (glycomics data)
Dataset S2 (Phyton scripts for CRT)

SI ***Appendix***

Supplementary Material and Methods

Determination of Ca^{+2} burst upon treatment with cell wall fractions

Eight-day-old liquid-grown apoaequorin-expressing seedlings (Col-0^{AEQ} and *agb1-2*^{AEQ}) were used for Ca^{2+}_{cyt} upon cell wall fractions treatment. Col-0^{AEQ} and *agb1-2*^{AEQ} plants (were grown in 24-well plates (~10 seedlings per well) under long day conditions (16 h of light) at 20-22ºC in liquid MS medium. Then, they were placed individually in 96-well plates in coelenterazine (PJK GmbH) and water and were incubated overnight in the dark. Luminescence was recorded with a Varioskan Flash Multimode Reader (Thermo Scientific) as described, after treatment with cell wall extracts (PEC1 and PEC2) from wild-type plants and cell wall mutants (1). *agb1-2*^{AEQ} lines were generated by crossing *agb1-2* with Col-0^{AEQ} plants, and homozygous *agb1-2*^{AEQ} plants were selected by measuring the luminescence emitted after the addition of 2 M $CaCl_2$ to leaf discs from three-week-old plants and by allele-specific PCR amplification to confirm *agb1-2* background (2, 3).

Immunoblot analysis of MAPK activation

Twelve-day-old seedlings (n = 10) grown on liquid MS medium in 24-well plates were treated with water (mock) or PEC1 fractions (heat-treated at 121 ºC for 20 min; 50 ng/µl) from wild-type plants (Col-0) or cell wall mutants. Seedlings were harvested in liquid nitrogen at 0 (before treatment), 10 and 20 minutes. Protein extraction and detection of activated MAPKs using the Phospho-p44/42 MAPK (Erk1/2) (Thr202/Tyr204) antibody (Cell Signaling Technology) were performed as described (1). Phosphorylated MAPKs bands were detected with the iBright CL1000/FL1000 imaging system (Thermo Fisher Scientific) and MAPKs bands intensity was determined using the software of the equipment and following manufacturer instructions.

Determination of Ca^{+2} in cell wall fractions

Total free Ca^{2+} in PEC1 and PEC2 cell wall fractions was determined by atomic absorption spectrophotometry (4) in the Analytical Chemistry Unit of the Escuela Técnica Superior de Ingeniería Agronómica, Alimentaria y de Biosistemas (Universidad Politécnica de Madrid). Equipment was calibrated with 1, 5 and 3 ppm standards. PEC1 and PEC2 fractions were diluted to 1 mg fraction/mL and centrifuged for 5 minutes at full speed. Aqueous supernatants were directly measured, whereas pellets containing materials in suspension total Ca^{2+} were first acid-digested with 14 cm^3/g 15 M HNO_3 and 6 cm^3/g 27 M HCl followed by a digestion at 120 ºC for 2 h in Teflon bombs in an oven.

Plant tolerance to desiccation assays

Three-week-old soil-grown plants (n=8) were restricted completely from irrigation for 21 days and then wilted plants were re-watered for 7 days and the number of plants that survived to the stress and recovered their developmental phenotype was scored. These experiments were performed four times.

Mathematical Modelling

The description of the mathematical analyses performed is shown in the schema of *SI Appendix*, Fig. S16, and can be divided into two interrelated tasks that share a set of analytical steps. These common analyses were initiated with the experimental data on susceptibility to pathogens, fitness and tolerance to desiccation of *cwm* and wild-type plants (Fig. 1 and Fig. 2A, B, and *SI Appendix* Fig. S3 and Fig. S7A). For each of these 6 response variables (resistance to 3 pathogens, 2 fitness parameters and plant desiccation tolerance) a different two-way ANOVA model was fit for each ecotype (Col-0, Ws-0, or La-*er*), considering the genotype as the factor/effect of interest, but also introducing a second factor/effect to allow collecting together the data from a number of similar experiments (2 independent experiments in the case of pathogen resistance, 3 for fitness and 3 for tolerance to desiccation). No interaction was initially considered between both factors/effects, rendering the experiment effect as a block. These initial analyses aim to confirm the known significance of the genotype factor/effect, and to estimate the least squares means (or LS means) of each response in each genotype. These LS means provide a single estimation of the average response level (e.g. mean disease rating for both *Pc* and *Rp*, mean conidiospores/mg fresh weight for *Ha*, mean seed yield in mg/plant, mean rosette fresh weight/biomass in g, and mean survival rate (%) after desiccation) for each genotype under consideration, controlling the effect of the experimental differences due to the unbalanced nature of the designs.

The correlation analysis between mean biotic stress resistance, on one side, and mean fitness and desiccation tolerance, on the other side, was then performed on this intermediate set of 21 genotypes. To this aim, the percentage ratio of each genotype LS mean to that of the corresponding ecotype wt was obtained for each response variable. These susceptibility ratios allowed expressing the average response information of each genotype in relation to that of the reference wt, as well as bringing the information of different ecotypes to a similar scale. A linear model was then fit for each combination of the log-transformed biotic susceptibility ratios with the fitness and abiotic susceptibility ratios to analyze their correlations (see Fig. 2 C, D and *SI Appendix*, Fig. S7; for the fitted equations, *R*-squares and *p*-values; *SI Appendix*, Figure S16). As a consequence of the logarithmic transformation of the biotic susceptibility ratios, the *x*-axes in these figures range between 0 (smaller susceptibility) to 5 (greater susceptibility). The SAS software has been used to implement the previous analyses (*glm* and *corr* procedures).

Next, a paired comparisons analysis on the mentioned LS means was done, and experimental data on glycomic response of cell wall mutants' fractions (Fig. 3C and Dataset S1) was then incorporated to obtain predictive classification models (correlating wall composition with disease resistance and fitness phenotypes. More specifically, on the basis of the *p*-values of the one-sided Dunnett tests (α = 0.05), each genotype was assigned a class (e.g. a categorical valuation) for each response, which represent its status in such feature compared to the wt: either a plant has a similar performance to the wt (class *E*), or the plant performs significantly better than the wt (class *B*) or the plant performs significantly worse than the wt (class *W*). A supervised classification methodology was then applied in order to correlate wall composition with biotic stress resistance, fitness, and desiccation tolerance phenotypes as represented by the mentioned classes *E*, *B* and *W*. The available data to describe wall composition was given by 32 glycomic measurements of a set of 155 antibodies (for each cell-wall fraction) performed for 11 different genotypes of the Col-0 ecotype (Fig. 3) Thus, the objective of the present analysis was to uncover and generalize the potential relationships between the set of glycomic responses (that therefore act as independent or explanatory variables) and the classes that describe the performance of the genotypes (acting as dependent variables), for each of the studied features, to explain the different phenotypical status of the genotypes in the core set, and to generalize such patterns in order to provide a mathematical model exhibiting an adequate predictive accuracy.

Many statistical and machine learning techniques are available to fit supervised classification models, as for instance linear discriminant analysis, logistic regression, random forest or classification trees. In the present analysis we have chosen this last technique (classification trees) since it constitutes a well-known, standard classification methodology with almost no statistical assumptions that provides interpretability of the resulting models, automatic independent variables selection, and an adequate predictive capability for the purposes of the present study. More specifically, the particular classification tree algorithm applied, known as CRT (or CART, from Classification and Regression Tree), is a non-parametric technique that makes no assumptions on the distribution of the data and the variability and balance between classes. The bases of the CRT algorithm are both the identification of independent variables (in this case, cell-wall antibodies) and the definition of cut-points on these variables' values that allow separating in different branches of a tree the instances belonging to different classes. In this way, departing from an initial *root* node containing all the instances of the training sample, CRT tries to obtain purer or more homogenous nodes (in terms of their class composition) by computing the reduction of the node's impurity allowed by each of the available independent variables. Given a set of n classes, each appearing in a given node with a relative frequency f_i, $i = 1,\ldots,n$, CRT computes the node's impurity through the Gini Index

$$I_G = \sum_{i=1}^{n} f_i (1 - f_i).$$

Thus, from a given node, CRT selects both the variable and its cut-point that allow the greatest impurity reduction from the “father” node to the “child” nodes. CRT is based on binary branching, e.g. only two child nodes are produced from each father node being split. The main inconvenient of the CRT methodology is its tendency to produce overfit, e.g. to provide classification models that generalize not only the right, essential patterns or relationships between the explanatory variables and the classes, but also the potential noise that the specific data used to construct (or train) the model may contain. When overfit is present, the obtained model tends to provide excellent results on the training sample, but quite worse results on unseen instances not used in the construction of the trees, what is usually called a validation or test sample. To avoid overfit, we force the tree growth process to stop after the first split of the root node, and at the same time we carry out a demanding cross-validation process in order to get a precise estimation of the actual model’s accuracy. Therefore, the obtained tree is composed of just the root node and two leaves, defined through a cut-point value for a single explanatory variable. In this way, we focus on uncovering just the main, most discriminant antibody for separating genotypes exhibiting a different phenotypical performance (see Fig. 4, SI *Appendix*, Fig. S10-S12). Once this was done, we focused on estimating the actual accuracy of the obtained model by conducting a 10-fold cross-validation process, e.g. the available data is randomly divided in 10 similar-size parts, and then similar trees are trained using 9 of these parts, leaving the remaining part to be used as a test sample (as these instances are not used in the tree fitting step). This step is carried out 10 times, each time using a different part as test sample. The proportion of test-sample instances correctly classified throughout this 10-steps process provides an estimation of the original model’s accuracy. To obtain a more robust estimation and average out the dependence on the random division step, the whole 10-fold cross-validation process was replicated 100 times, each time using a different random division of the available data in 10 parts. The mean and standard deviation of these 100 10-fold cross-validation estimations at each addressed classification task are reported in Fig. S10*B* and Table S1. Both the CRT models fit and their cross-validation were performed using Python *scikit-learn* library.

Glycome analysis by Enzyme-Linked ImmunoSorbent Assay (ELISA)

mAbs were obtained as hybridoma cell culture supernatants either from laboratory stocks (CCRC series, JIM series, MAC series; available from CarboSource [http://www.carbosource/net]) or from Plant Probes (LM series, PAM1 [http://www.plantprobes.net/]) unless otherwise indicated. A detailed list of all mAbs included in this study showing the immunogens used to develop them, their isotype, and the cell wall

polysaccharide class they primarily recognize is provided in Dataset S1. The experimental protocol previously described (5) was performed with minor modifications. In brief, flat-bottom 96-well ELISA plates (Nunc 439454 from Thermo Fisher Scientific or 2507 Costar from Corning Life Sciences), we used to apply cell wall mutant fraction polysaccharides (50 μL of 10 μg mL−1 in deionized water per well, or deionized water for controls) and then plates were dried to the well surfaces by evaporation overnight at 37°C. The plates were blocked with 200 μL of 1% (w/v) instant nonfat dry milk (Carnation) in Tris-buffered saline (50 mm Tris-HCl, pH 7.6, containing 100 mm sodium chloride) for 1 h. All subsequent aspiration and wash steps were performed using an ELx405 microplate washer (Bio-Tek Instruments). Blocking agent was removed by aspiration, and 50 μL of undiluted hybridoma supernatant of each antibody were added to the well and incubated for 1 h at room temperature. Supernatant was removed and wells were washed three times with 300 μL of 0.1% (w/v) instant nonfat dry milk in Tris-buffered saline (wash buffer). Peroxidase-conjugated goat anti-mouse IgG or goat anti-rat IgG antibodies (Sigma-Aldrich), depending on the primary antibody used, was diluted 1:5,000 in wash buffer, and 50 μL were added to each well and incubated for 1 h. Note that the secondary antibodies used in this study are generated against whole immunoglobin molecules and thus bind to several isotypes of primary antibodies, including IgGs, IgMs, and IgAs, according to the manufacturers. Wells were then washed five times with 300 μL of wash buffer. 3,3′,5,5′-Tetramethylbenzidine substrate solution (Vector Laboratories) was freshly prepared according to the manufacturer's instructions, and 50 μL were added to each well. After 20 min, the reaction was stopped by adding 50 μL of 0.5 n sulfuric acid to each well. The OD of each well was read as the difference in A450 and A655 using a model 680 microplate reader (Bio-Rad). The reading from each test well was subtracted from that of a control well on the same plate that contained the same primary and secondary antibodies but no immobilized polysaccharide. This experiment was repeated 3 times with 2 independent biological replicates.

Locus	Allele Tested	Allele Mutant Line	Type of Mutation	Ecotype	Protein Function	Reference
At1g12840	***det3-1***	CS6160 (T=> A in 1st intron)	HM	Col-0	Subunit C vacuolar H+ATPase (V-ATPase)/ De-ETiolated 3 (DET3)	Luo et al., 2015<br>Delgado-Cerezo et al., 2012
	det3-2	SAIL_517_E02	LFM			
At1g20850	***xcp2-1***	SALK_057921.45.15.x	HM	Col-0	Papain type cystein endopeptidase/ Xylem cysteine protease 2 (XCP2)	Funk et al., 2002<br>Avci et al., 2008<br>Zhang et al., 2014
	xcp2-2	SALK_010938.56.00.x	LFM			
At1g23170	***at1g23170-1***	SALK_046821.54.50.x	LFM (Fig S2)	Col-0	Protein of unknown function DUF2359; Transmembrane protein	Coates, 2003
	at1g23170-2	SALK_056368.55.50.x	LFM			
At1g27440	***irx10-1***	SALK_055673	LFM	Col-0	Glycosyl transferase family 47	Persson et al., 2005<br>Brown et al., 2005
At1g56330	*sar1b-4*	FLAG_141E05	LFM	Ws	Sec23a/Small GTP-binding protein/ ARF-like GTPase family (SAR1b)	Zeng et al., 2015
At1g56340	*crt1-2*	FLAG_048A05	LFM	Ws	Calreticulin 1 (CRT1)	Christensen et al., 2010
At1g65580	***fra3-1***	SAIL_253_E02	HM	Col-0	Type II inositol polyphosphate 5-phosphatase/FRAgile Fibre 3 (FRA)	Zhong et al., 2004
At1g69530	***exp1-1***	FLAG_401A10	LFM (Fig S2)	Ws	Expansin 1 (At-EXP1)	Esmon et al., 2005
At1g70770	***at1g70770-1***	SALK_023673.43.50.x	HM (Fig S2)	Col-0	Protein of unknown function DUF2359, Transmembrane Protein	Schmidt et al., 2007
	at1g70770-2	SALK_110286	LFM			
At1g75500	*wat1-1*	SALK_001389	LFM	Col-0	Walls Are Thin 1 (WAT1)/Nodulin21	Ranocha et al., 2010<br>Denancé et al., 2013
At2g02120	*pdf2.1-2*	FLAG_441H10	LFM (Fig S2)	Ws	Plant defensin protein, PDF2.1	Siddique et al., 2011
At2g27040	***ago4-1t***	SALK_007523	HM (Fig S2)	Col-0	ARGONAUTE 4 (AGO4)	Agorio & Vera, 2007
At2g38080	*irx12-1*	SALK_051892	LFM	Col-0	Laccase 4/IRregular Xylem 12 (IRX12)	Brown et al., 2005<br>Yi Chou et al., 2018
At3g10740	*araf1-1*	FLAG_091G07	LFM	Ws	α-L-ARAbinoFuranosidase 1 (ARAF1/ASD1)	Montes et al., 2008
At3g16920	*ctl2-1*	SALK_055713.38.85.x	LFM	Col-0	ChiTinase-Like protein 2 (CTL2)	Sánchez-Rodríguez et al., 2012
At3g53210	*mtn21-1*	FLAG_258G08	LFM	Ws	Nodulin MtN21 family protein	Busov et al., 2004
At3g54920	*pmr6-1*	CS6354 G140D	HM	Col-0	Pectato Lyae Like/Powdery Mildew Resistance 6 (PMR6)	Vogel et al., 2002<br>Wang et al., 2017
At4g02380	***sag21-1***	SALK_099663.54.70.x	HM	Col-0	Senescence Associated Gene 21 (SAG21)	Salleh et at., 2012
At4g15160	***at4g15160-1***	SALK_007014	HM (Fig S2)	Col-0	Protease inhibitor/Lipid Transfer protein (LTP)	n/a
At4g18780	***irx1-6***	W114Stop (EMS, see reference)	LFM (truncated protein)	Col-0	Cellulose Synthase 8 (CESA8)/IRregular Xylem 1 (IRX1)	Taylor et al., 2000<br>Hernández-Blanco et al., 2007
At4g34460	*agb1-1*	CS3976 (G=> T splice 1st exon)	LFM	Col-0	Arabidopsis heterotrimeric G Protein Β subunit 1 (AGB1)	Delgado-Cerezo et al., 2012<br>Llorente et al., 2005
At4g37770	***acs8-2***	SALK_066725	HM	Col-0	1-aminocyclopropane-1-carboxylate synthase-like protein 8 (ACS8)	Zhang et al., 2018
	acs8-3	SAIL_102_E05.v1	LFM			
At5g04370	***namt1-1***	SALK_001690.56.00.x	LFM	Col-0	Nicotinate MethylTransferase 1 (NaMT1)	Wu et al., 2018
	namt1-2	SAIL_300_D11	LFM			
At5g15630	***irx6-1***	FLAG_248B03	LFM	Ws	COBRA-LIKE 4 (COBL4)/IRregular Xylem 6 (IRX6)	Brown et al., 2005<br>Delgado-Cerezo et al., 2012
At5g17420	***irx3-1***	CS104 W859Stop	LFM (truncated protein)	La-*er*	Cellulose Synthase 7 (CESA7/MUR10) IRregular Xylem 3 (IRX3)	Turner & Somerville, 1997<br>Hernández-Blanco et al., 2007
At5g18650	*miel1-1*	SALK_097638	LFM	Col-0	MYB30-interacting E3 ligase (MIEL1)	Marino et al., 2013
At5g26120	*araf2-1*	SALK_033343	LFM	Col-0	α-L-ARAbinoFuranosidase 2 (ARAF1/ASD2)	Fulton & Cobbett, 2003
At5g49720	***irx2-1***	P250L (EMS, see reference)	HM	La-*er*	KORRIGAN1 (KOR1), IRregular Xylem 2, β,1-4 endoglucanase	Szyjanowicz et al., 2004<br>López-Cruz et al., 2014
At5g51890	***at5g51890-1***	SALK_086448.49.80.x	LFM (Fig S2)	Col-0	Peroxidase 66 (PRX66)	Sato at al., 2006
At5g54690	*irx8-1*	SALK_014026	LFM	Col-0	Galacturonosyltransferase (GAUT1)/IRregular Xylem 8 (IRX8)	Persson et al., 2007;
At5g58600	*pmr5-1*	CS6579 W265Stop	HM (truncated protein)	Col-0	Pectin acetyltransferase/Powdery Mildew Resistance 5 (PMR5)	Vogel et al., 2005<br>Chiniquy et al., 2019
At5g60340	*aak6-1*	SALK_015289	LFM	Col-0	Adenylate Kinase isoenzyme 6 homolog (AAK6)	Slovak et al., 2020
At5g62920	*arr6-2*	SALK_008866.55.00.x	HM	Col-0	Arabidopsis Response Regulator 6 (ARR6)	Bacete et al., 2020
	arr6-3	SALK_133123.21.20.x	LFM			
At5g63670	*spt4-1*	FLAG_460C02	LFM (Fig S2)	Ws	Transcription elongation factor SPT4 homolog 2	Dürr et al., 2014

Figure S1. *Arabidopsis thaliana m*utants used in the disease resistance and fitness analyses. Gene locus, mutant allele tested and type of mutation (loss of function mutation (LFM) or hypomorphic mutation (HM) alleles) are described. In bold are indicated mutant alleles analyzed for fitness parameters (biomass and seed yield; Fig. 2). Additional mutant alleles tested in the disease resistance against *Pc* (Fig S4) are underlined. Reference of the mutant lines used are showed (T-DNA insertion lines or EMS-derived mutants with the indication the amino acid change or exon splicing changes). RT-PCR of those mutants characterized in this work are shown in Fig. S2. References describing previously a function of the indicated gene/protein in cell wall biogenesis/composition are indicated in green, in disease resistance are marked in red and if both phenotypes (cell wall/disease resistance) are indicated in blue.

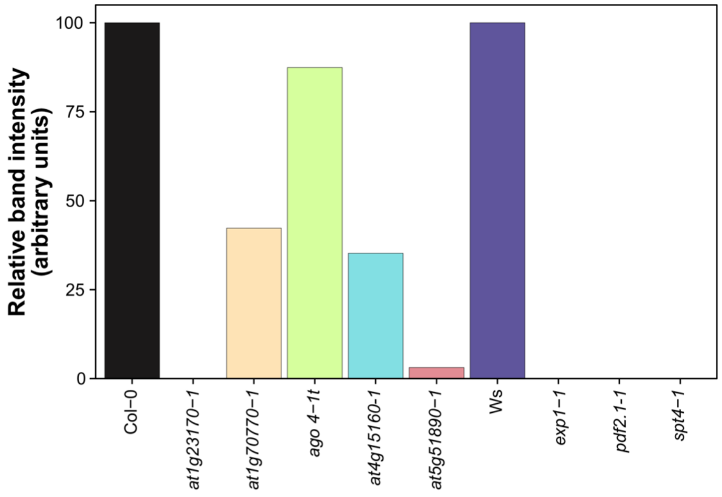

Figure S2. RT-PCR expression of genes in *Arabidopsis* mutants used in the disease resistance analyses. Expression of the indicated genes were determined in four-week old wild-type plants (Col-0 and Ws) and in the indicated mutants by extracting total RNA and performing RT-PCR using the oligonucleotides indicated in Table S3. PCR amplification bands were quantified using Fiji-J and the expression of the genes in Col-0 and Ws was set to 100% and the values of RT-PCR amplifications (n=2) in the mutants determined and represented.

Locus	Mutant allele	*P. cucumerina*		*R. pseudosolanacearum*		*H. arabidopsidis*	
		Disease Rating (0-5)	±SD	Disease Rating (0-4)	± SD	Spores/ mg fresh weight	± SD
Ecotype	**Col-0**	2.1	0.06	2.27	0.03	576.74	30.57
Ecotype	**Ws**	2.07	0.11	2.17	0.06	1661.64	122.65
Ecotype	**La-*er***	2.07	0.12	3.02	0.23	969.31	172.52
At1g12840	*det3-1*	1.77	0.13	0.01	0.15	227.74	38.24
At1g20850	*xcp2-1*	2.21	0.13	1.48	0.15	0	63.18
At1g23170	*at1g23170-1*	2.26	0.12	2.45	0.15	278.09	63.18
At1g27440	*irx10-1*	2.06	0.16	0.18	0.19	508.09	38.24
At1g56330	***sar1b-4***	2.43	0.1	2.31	0.3	1280.9	186.8
At1g56340	***crt1-2***	2.67	0.23	2.01	0.23	1455.16	396.45
At1g65580	*fra3-1*	0.95	0.19	0.08	0.15	258.59	38.43
At1g69530	***exp1-1***	1.91	0.26	1.98	0.12	1385.23	122.93
At1g70770	*at1g70770-1*	1.57	0.15	2.25	0.15	361.43	63.18
At1g75500	*wat1-1*	1.05	0.19	1.01	0.02	1418	202.8
At2g02120	***pdf2.1-2***	2.28	0.16	3.41	0.25	1782.9	203.19
At2g27040	*ago4-1t*	1.6	0.16	2.65	0.13	550.84	122.93
At2g38080	*irx12-1*	2.2	0.15	2.3	0.18	611.48	60.2
At3g10740	***araf1-1***	2.17	0.18	2.15	0.16	1438.77	170.44
At3g16920	*ctl2-1*	1.9	0.15	1.85	0.17	436.84	72.69
At3g53210	***mtn21-1***	1.24	0.15	2.02	0.25	2059.12	152.23
At3g54920	*pmr6-1*	1.96	0.15	2.05	0.2	496.07	72.69
At4g02380	*sag21-1*	1.76	0.18	2.74	0.15	201.29	63.18
At4g15160	*at4g15160-1*	2.07	0.13	2.37	0.15	0	63.18
At4g18780	*irx1-6*	1	0.1	0.05	0.13	477.75	38.24
At4g34460	*agb1-1*	4.8	0.2	2.15	0.15	679.11	72.69
At4g37770	*acs8-2*	1.93	0.18	1.18	0.15	304.09	63.18
At5g04370	*namt1-1*	1.64	0.13	2.23	0.15	395.48	63.18
At5g15630	***irx6-1***	1.14	0.16	2.38	0.16	1849.42	123.78
At5g17420	***irx3-1***	1.08	0.18	0.35	0.08	845.76	173.52
At5g18650	*miel1-1*	2.23	0.15	2.63	0.12	883.67	344.5
At5g26120	*araf2-1*	2.36	0.14	2.08	0.24	1509.72	301.8
At5g49720	***irx2-1***	1.84	0.16	3	0.23	951.39	172.52
At5g51890	*at5g51890-1*	1.61	0.17	2.35	0.15	430.73	30.57
At5g54690	*irx8-1*	1.97	0.18	2.47	0.18	441.33	72.69
At5g58600	*pmr5-1*	1.9	0.2	2.3	0.21	615.88	72.69
At5g60340	*aak6-1*	2.42	0.15	2.19	0.23	1668.48	222.5
At5g62920	*arr6-3*	1.81	0.1	3.23	0.2	401.26	38.24
At5g63670	***spt4-1***	0.61	0.1	1.95	0.28	3815.47	852.26
At3g48090	***eds1-1***		–		–	2570.99	325.35
	eds1-2		–		–	794.71	38.24
NahG	–		–		–	1020.58	122.9

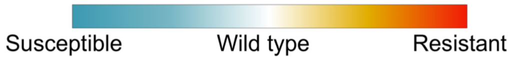

Figure S3. Disease resistance analysis of *Arabidopsis* cell wall mutants. Average disease resistance values (±SD) of wild-type plants and cell wall mutants in different backgrounds (black-Col-0; green-La-*er*; purple-Ws) to *P. cucumerina* BMM (DR from 1 to 5), *R. pseudosolanacearum* (GMI1000 strain for Col-0 and La-*er*, and RD15 for Ws; DR from 1 to 4), and *H. arabidopsidis* (Noco2 strain for Col-0, Ewma1 for Ws and Cala for La-*er;* conidiospore/mg plant fresh weight). Color-code of the corresponding columns indicates the level of the resistance phenotype, from susceptible (blue) to resistant (red) whose differences with wild-type plants (white) are statistically significant (Dunnett's test p≤0.05). White color of the columns values of the mutants means not statistically significant from wt plants. Genotypes used as control of resistance (*cr*) or control of susceptibility (*cs*) for the different pathogens are indicated with shaded cell. For *Hpa* La-*er* and Col-0 wild-type ecotypes were included as *cr* for Col-0 and La-*er*/Ws mutants backgrounds, respectively (conidiospores/mg fresh weight is equal to 0), and *NahG* plants (Col-0), *eds1-1* (Ws) and *eds1-2* (Col-0) alleles were used as *cs* for Col-0, Ws and La-*er* background, respectively.

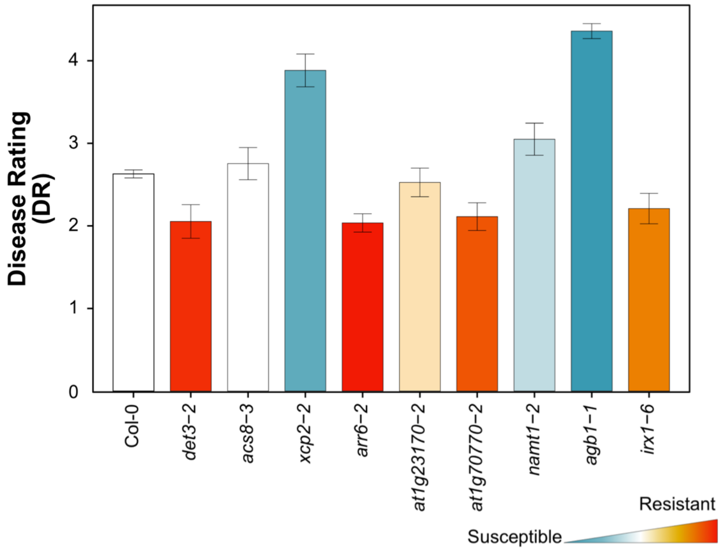

Figure S4. Disease rating (DR) of *cwm* second alleles inoculated with *P. cucumerina*. DR average (±SD) of wild-type (wt) plants (Col-0 background) and mutants at 7 days post inoculation (dpi) with the necrotrophic fungus *P. cucumerina*. DR varies from 0 (non-infected plants) to 5 (dead plants). Colored columns indicate significant differences compared with wt values (ANOVA non-balanced analysis and Dunnett's test $p \leq 0.05$). This is one representative experiment of the three performed that gave similar results (n = 10).

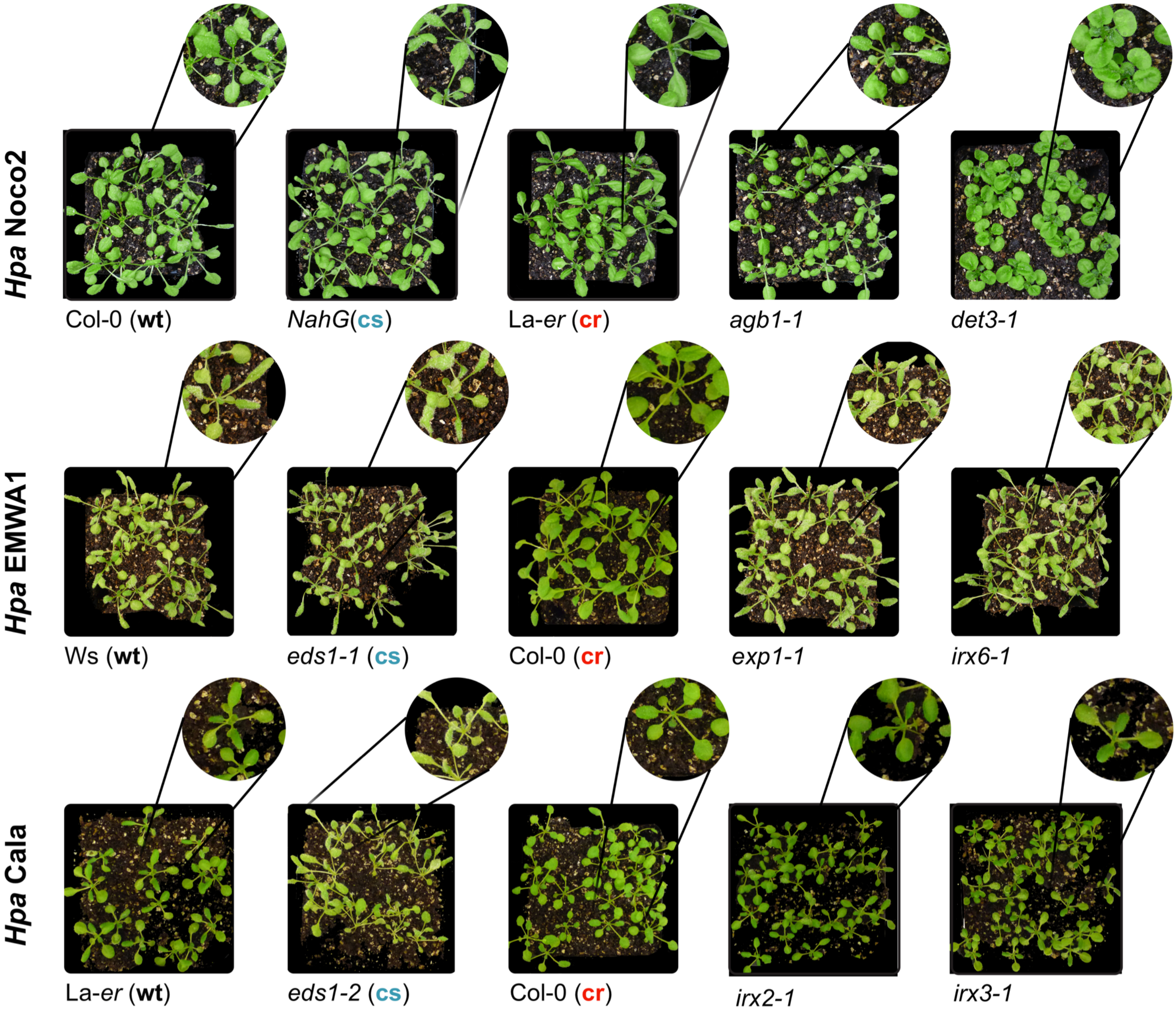

Figure S5. Macroscopic disease symptoms in representative genotypes inoculated with *H. arabidopsidis*. Pictures of Col-0, La-*er* and Ws wild-type plants, control of resistance (*cr*) and control of susceptibility (*cs*) genotypes, and some representative cell wall mutants at 7 days post inoculation with strains Noco2 (Col-0), Emwa1 (Ws) and Cala (La-*er*). La-*er* and Col-0 wild-type ecotypes were included as *cr* for those mutants in backgrounds Col-0 and La-*er*/Ws, respectively. *NahG* plants (Col-0), and *eds1-1* (Ws) and *eds1-2* (Col-0) mutant alleles were used as *cs* for Col-0, Ws and La-*er* background, respectively. In the picture magnifications, *Hpa* sporangiophores can be observed on surface of the inoculated leave. For additional details see Fig. S3.

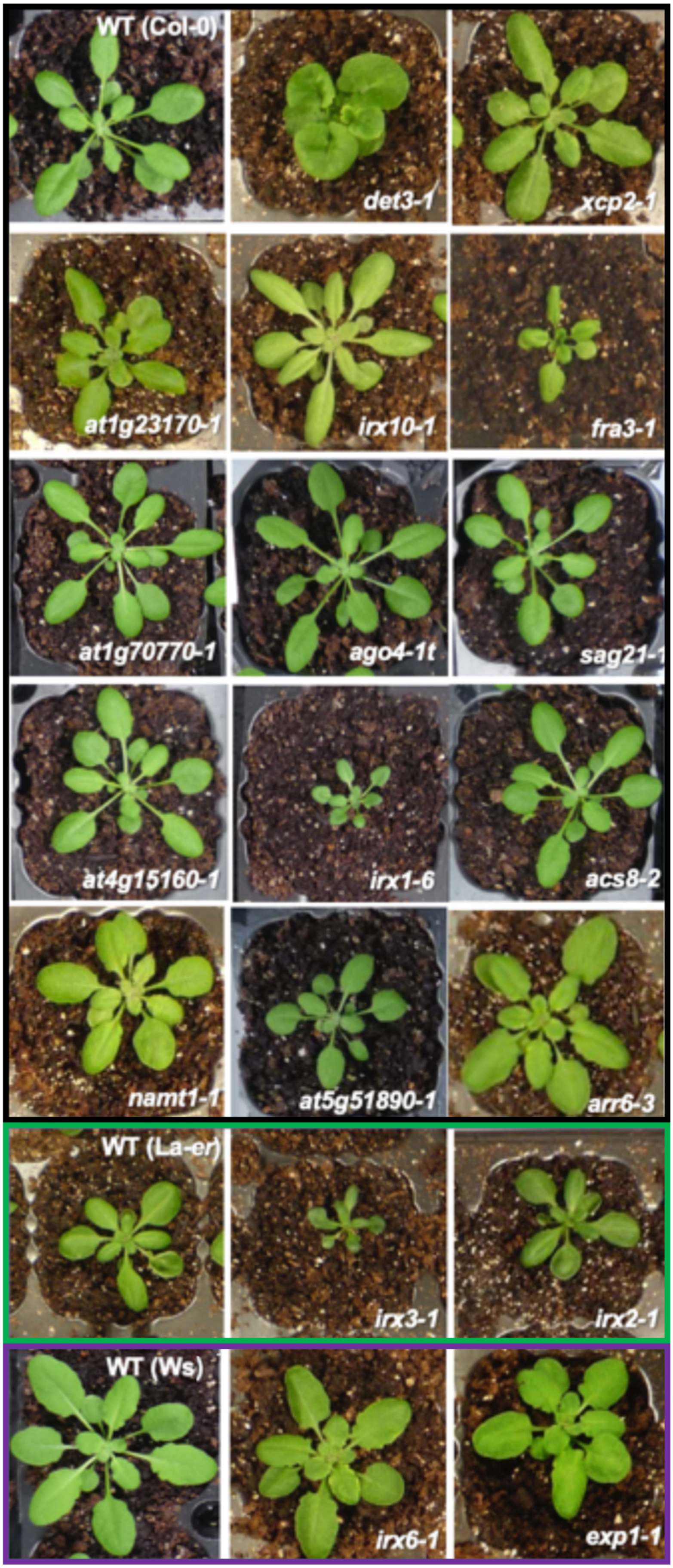

Figure S6. Developmental phenotypes of three-week old plants of *cwm* and wild-type ecotypes (Col-0, Ws and La-*er*). Plants were grown under short day conditions as described in Material and Methods.

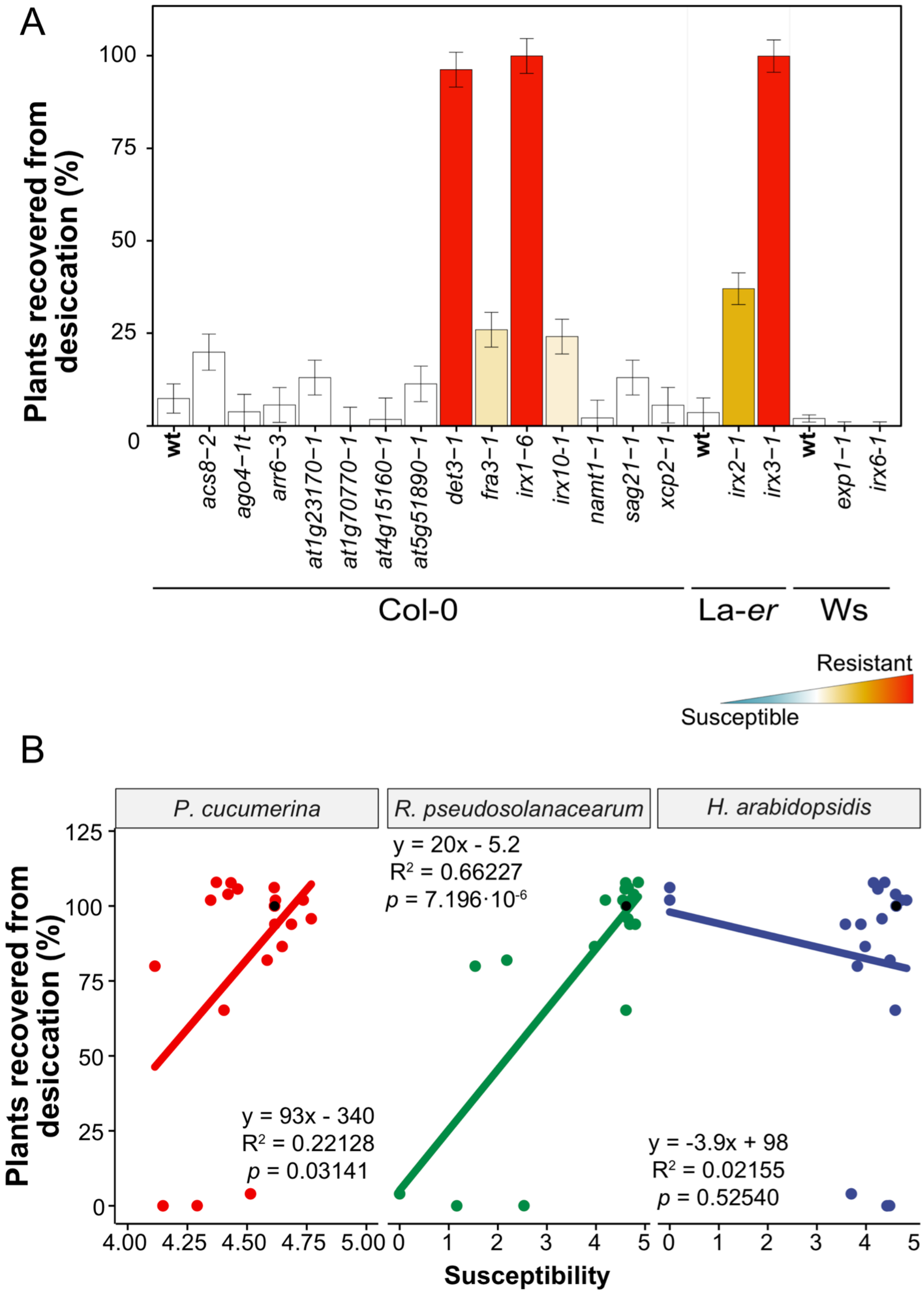

Figure S7. Desiccation tolerance of wild-type plants and cell wall mutants. *(A)* Percentage of plant survival after drought stress application for 21 days followed by plant re-watering. Data are the average of 10 plants. Columns color indicate significant differences (% of plant survival) compared with wild-type plants (wt) values (ANOVA non-balanced analysis, Dunnett´s test p≤0.05), with higher and lower values than wt indicated in red and blue, respectively. Mutant genotypes with lower values of survival than wt (blue color) were not identified. This is one representative experiment of the three performed that gave similar results. *(B)* Correlation analysis between desiccation tolerance and resistance to pathogens of 18 *cwm* mutants and wt plants. Average response information of each genotype (dot in the graph) is expressed in relation to that of the reference wild-type plant (black dot, value of 100% at the *y*-axes). Disease resistance ratios were log-transformed, and accordingly *x*-axes range from 0 (lower susceptibility) to 5 (greater susceptibility), with the wild-type plants situated at 4.72 = *ln* (1 + 100). A linear model was fitted for each combination and correlations determined. Fitted equations, R-squares and *p*-values are indicated in the insets of graphs. The *x*-axes of the figures involving *Pc* are enlarged in the 4-5 range for better visualization.

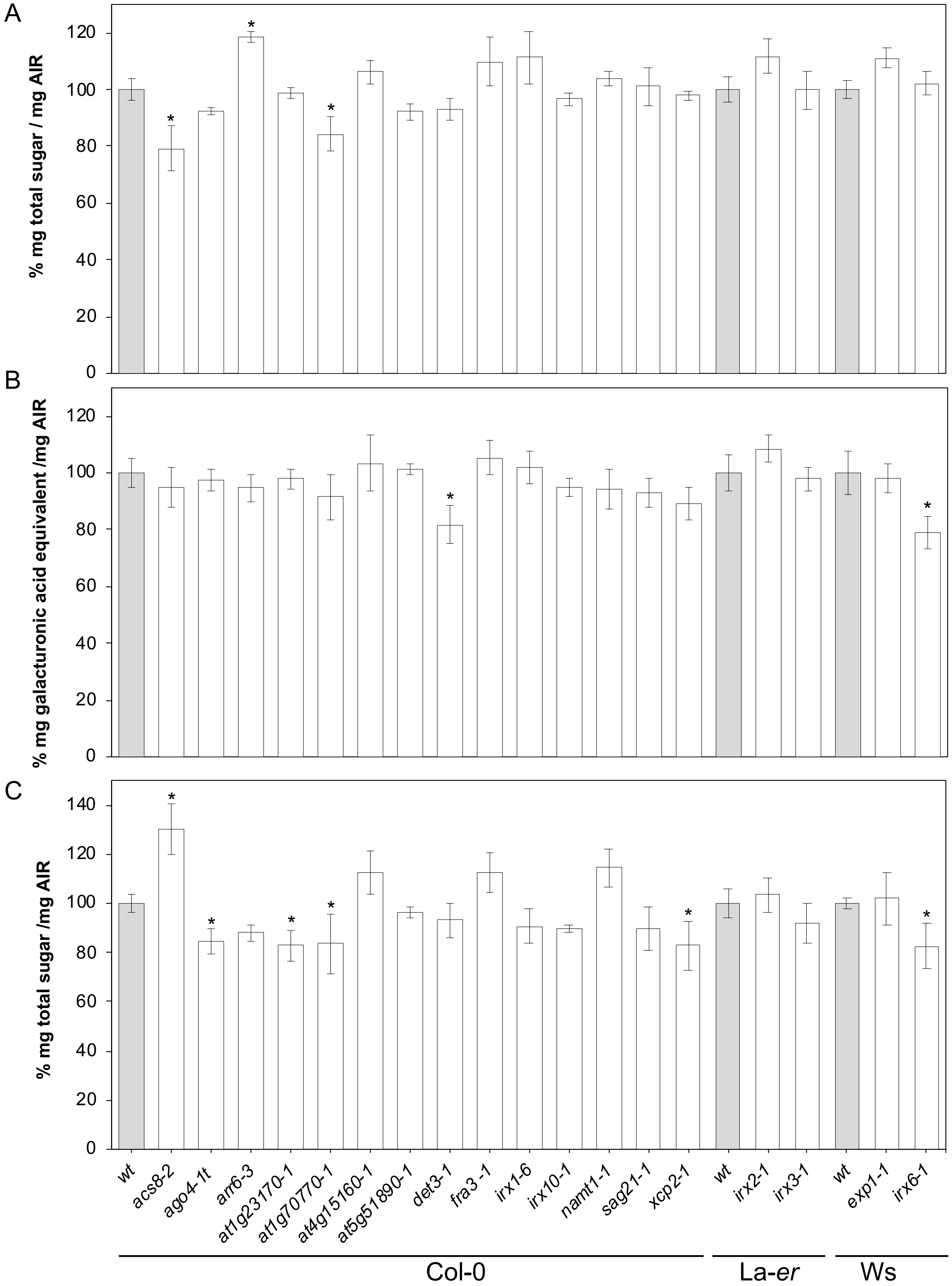

Figure S8. Cell wall biochemical composition of *Arabidopsis cwm* plants. (*A*) Total sugars quantification (% μg per mg of dry weight) in the non-cellulosic carbohydrate fraction from the cell walls of the mutants and their corresponding background plants (Col-0, La-*er* or Ws). (*B*) Total uronic acid (UA) content (% μg per mg of dry weight) from the cell walls of the indicated genotypes. (*C*) Cellulose content (% μg of total sugars per mg of dry weight). Data represent average values (± SE) of three independent experiments. Asterisks indicate mean values significantly different from wild-type plants (Student's *t*-test. $p < 0.1$; n > 10).

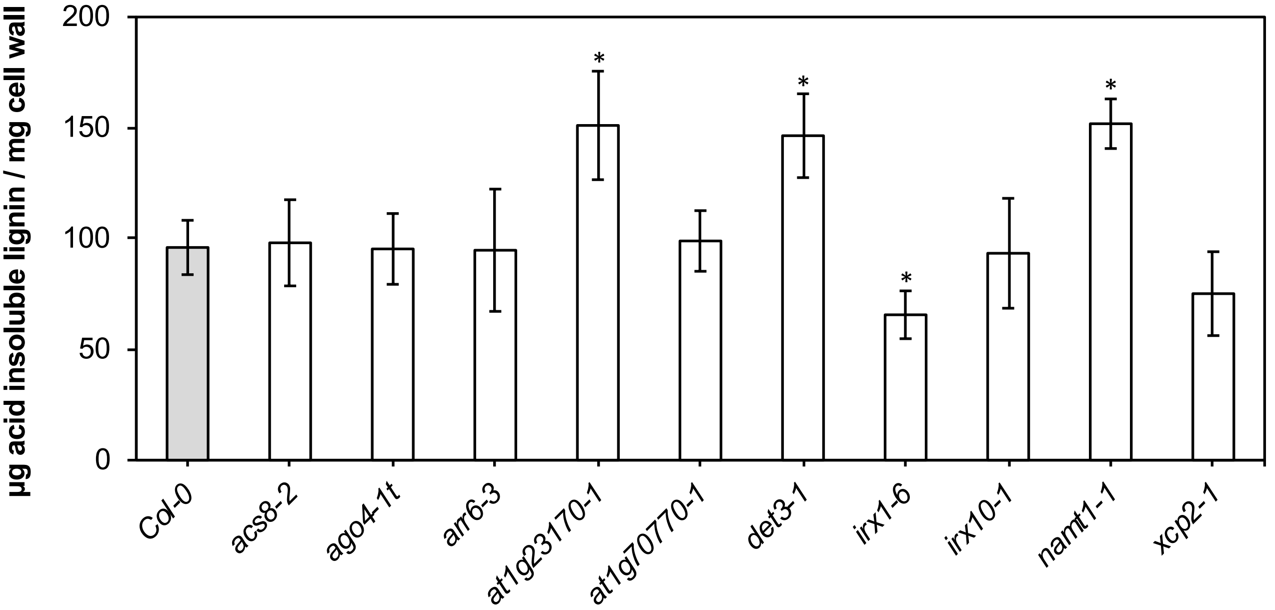

Figure S9. Total lignin content of *Arabidopsis* wild-type and *cwm* plants. Quantification of total lignin (µg acid insoluble lignin per mg cell wall) in the indicated mutants and their corresponding background plant (Col-0). Data represent average values (± SE) of three independent experiments. Asterisks indicate mean values significantly different from wild-type plants (Student's *t*-test. $p < 0.1$; n > 10).

A

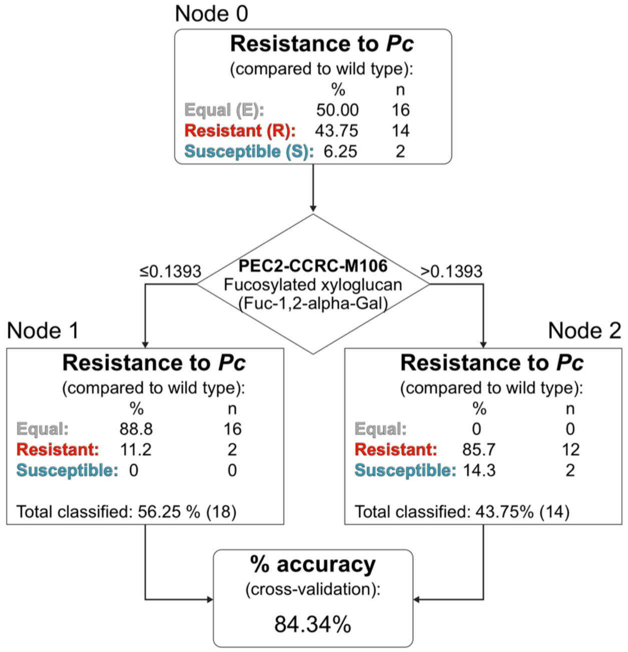

B

Phenotype	CW Fraction	Antibody	Abs. cut - off	Classification (compared to wt) R/H	E	S/L	Accuracy
Resistance to *P. cucumerina*	PEC2	CCRC_M106	≤ 0.1393	2	16	0	84.34%
			> 0.1393	12	0	2	
Resistance to *R. pseudosolanacearum*	PEC2	CCRC_M5	≤ 0.2990	10	0	2	83.88%
			> 0.2990	0	18	2	
Resistance to *H. arabidopsidis*	PEC2	CCRC_M106	≤ 0.1300	4	12	0	83.43%
			> 0.1300	16	0	0	
	HEC1	CCRC_M174	≤ 0.0005	0	7	0	82.96%
			> 0.0005	20	5	0	
Rosette biomass	PNS	CCRC_M22	≤ 0.4195	0	13	0	87.31%
			> 0.4195	0	3	16	
Seed production	HEC1	CCRC_M175	≤ 0.0052	0	21	0	87.62%
			> 0.0052	2	1	8	
	PEC1	CCRC_M170	≤ 0.0367	1	20	0	85.05%
			> 0.0367	1	2	8	
Tolerance to desiccation	HEC2	JIM 101	≤ 0.0040	2	24	0	83.16%
			> 0.0040	6	0	0	

Figure S10. Predictive CRT model correlating wall composition and disease resistance/fitness/desiccation phenotypes of *Arabidopsis* cell wall mutants. (*A*) Scheme of the CRT model obtained in the analysis of the correlation between resistance to *P. cucumerina* and cell wall epitopes. The tree model obtained with antibody CCRC-M106 (fucosylated xyloglucan) in PEC2 fraction of *cwm* and wild-type plants is shown. (*B*) Summary of the most relevant CRT models obtained for the different variables (resistance to pathogens, fitness and desiccation tolerance). For each variable, the CRT-selected antibodies detecting epitopes of some cell wall extracts as well as their absorbance cut-points are indicated (see A). The number of observations (*cwm* and wild-type plants) of each disease resistance/fitness/desiccation phenotype (resistant/higher (R/H), equal (E) or susceptible/lower (S/L) than wild-type plants) verifying either side of the cut-points is also reported. The predictive accuracy (% accuracy) of each model is estimated as the percentage of correct classifications in 100 replications of a 10-fold cross-validation process.

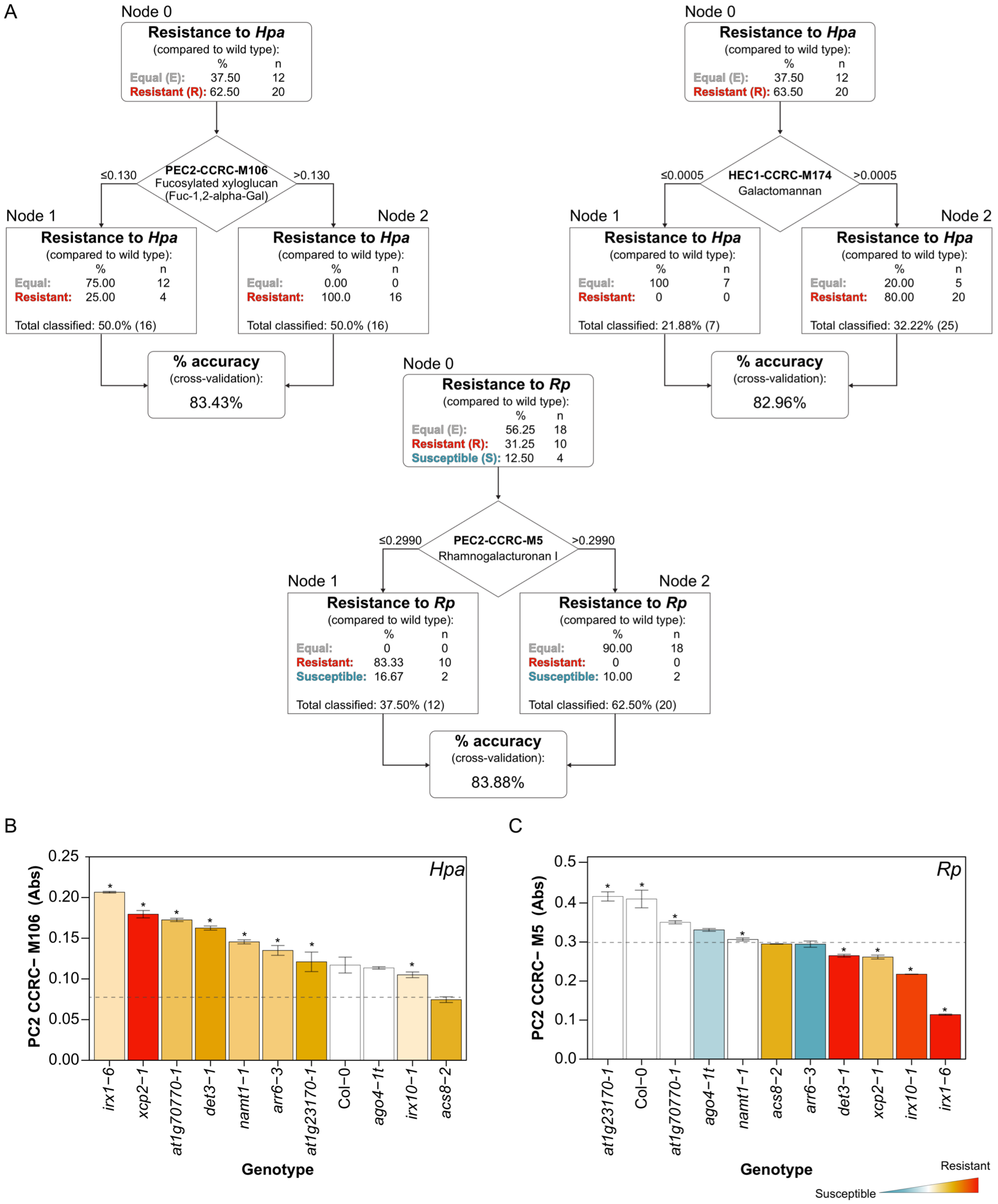

Figure S11. Predictive CRT model correlating wall composition and disease resistance/fitness phenotypes of *Arabidopsis* cell wall mutants to *H. arabidopsidis and R. pseudosolanacearum*. *(A)* Scheme of the CRT models obtained in the analysis of the correlation between resistance to *H. arabidopsidis* or *R. pseudosolanacearum* and cell wall epitopes. The tree models obtained with antibodies in cell wall fractions of cell wall mutants and wild-type plants are shown. *(B)* Biological validation of CRT results with cell wall mutants from 6 clusters analyzed. The absolute value (average ±SD) of the epitope signal detected by the antibody are shown (n = 3). The color code of the column indicates the resistance level of the corresponding mutant, from red (resistant) to blue (susceptible) in comparison with wild-type (wt) disease resistance level (in white) (see Fig. 1). The absorbance cut-point value for considering a mutant as resistant, as determined by CRT, is indicated by the dotted lines. *Arabidopsis* cell wall mutants that fulfill the CRT model are marked with an asterisk.

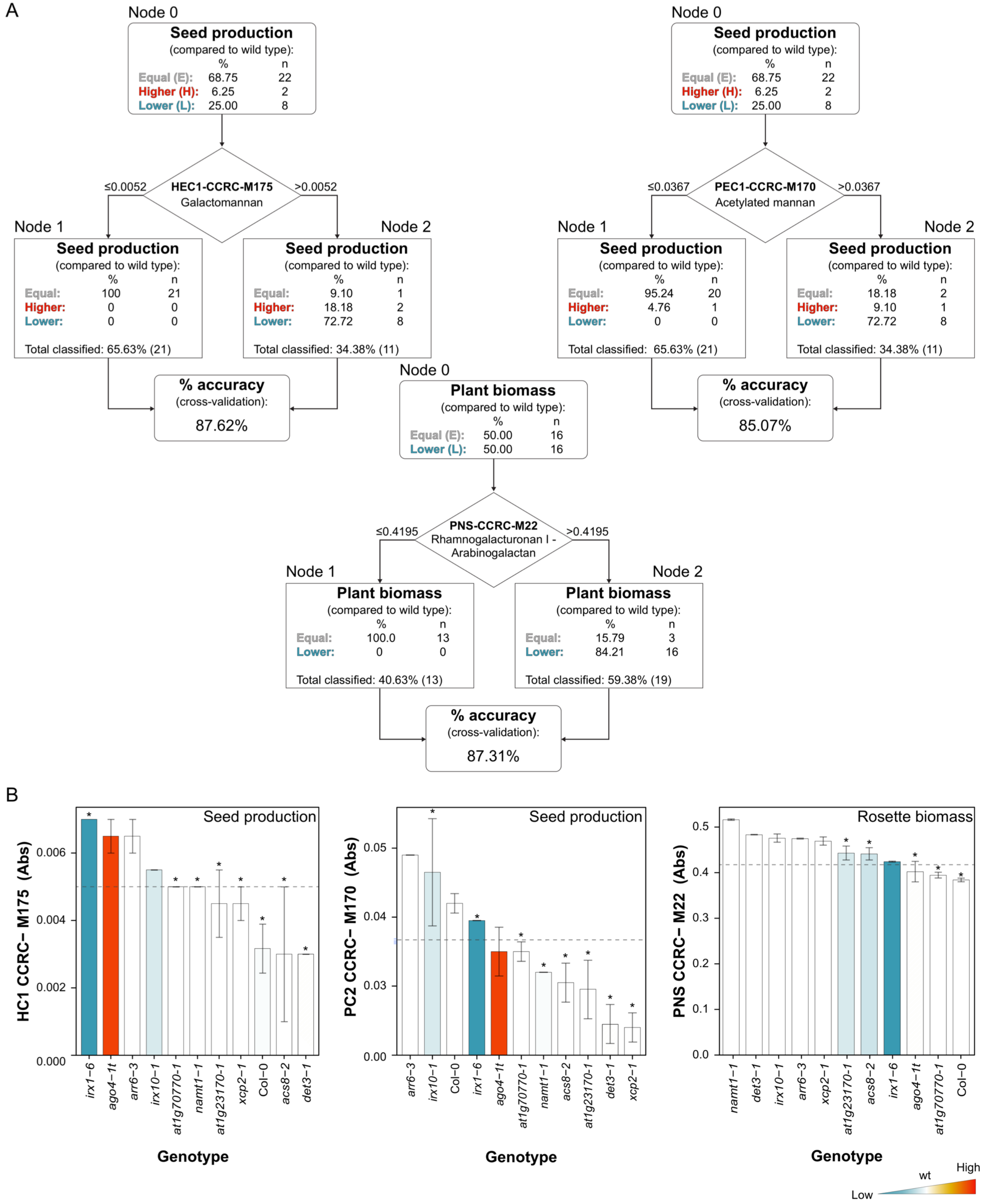

Figure S12. Predictive CRT model correlating wall composition and fitness phenotypes of *Arabidopsis* cell wall mutants. *(A)* Predictive CRT model correlating wall composition and fitness phenotypes of *Arabidopsis* cell wall mutants. *(A)* Scheme of CRT models obtained in the analysis of the correlation between fitness and cell wall epitopes. The trees models obtained with antibodies in cell wall fractions of cell wall mutants and wild-type plants are shown. *(B)* Biological validation of CRT results with the cell wall mutants analyzed. The absolute value (average ±SD) of the epitope signal detected by the antibody are shown (n = 3). The color code of the column indicates the level of the fitness value corresponding to the cell wall mutant, from red (enhanced values) to blue (reduced values) in comparison with wild-type (wt) values (in white) (see Fig. 2A,B). The absorbance cut-point value for considering a cell wall mutant as matching the CRT model is indicated by the dotted lines. *Arabidopsis* cell wall mutants that fulfill the CRT model are marked with an asterisk.

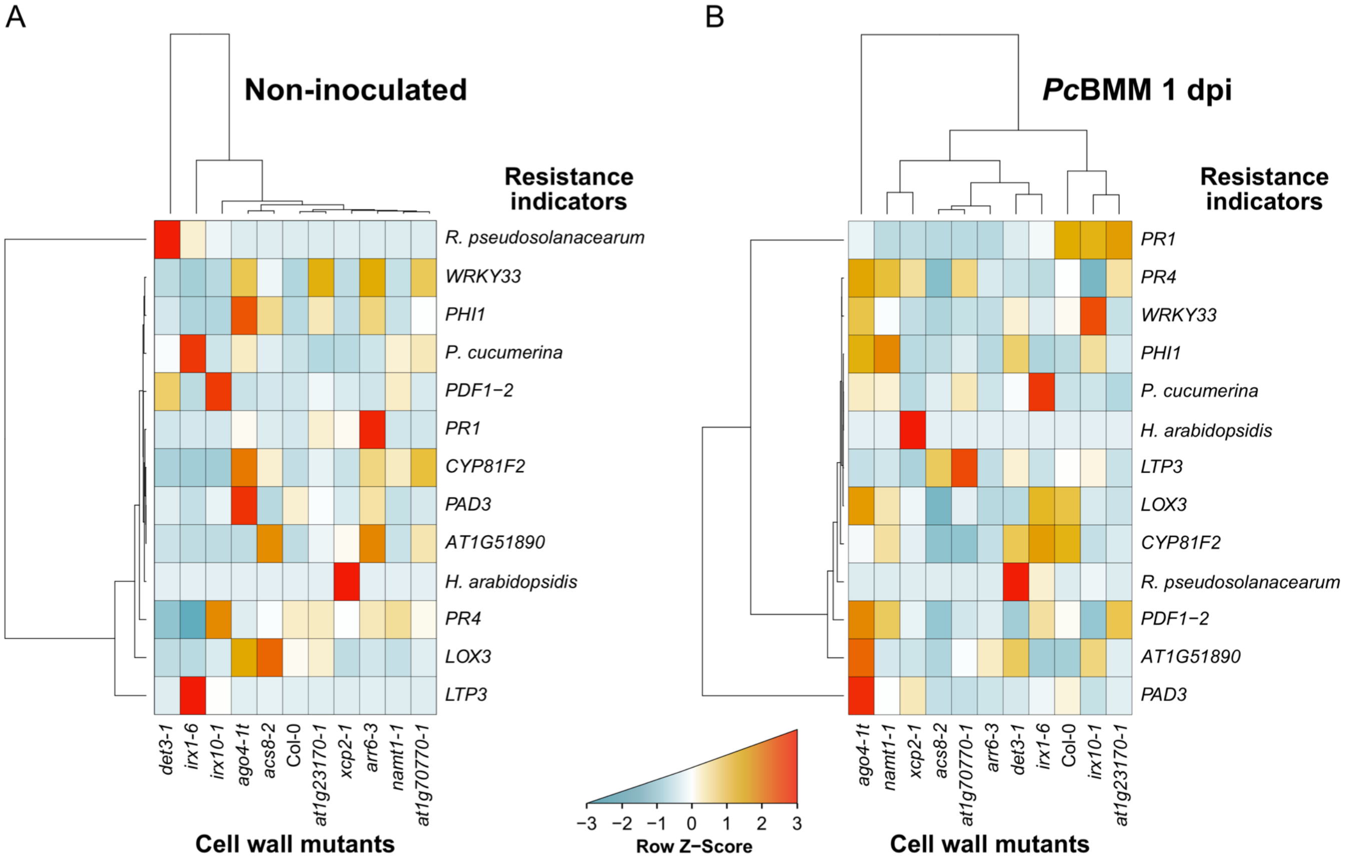

Figure S13. Clustering of the expression pattern of canonical defensive genes and disease resistance phenotypes in wild-type plants and cell wall mutants and their disease resistance phenotypes. (*A*) Clustering of disease resistance phenotypes and expression levels (determined by qRT-PCR) of defensive and MAMP-induced genes in non-inoculated three-week old plants from the indicated genotypes. (*B*) Relative expression (*PcBMM*/Mock at 1-day post inoculation, dpi). Clusters were computed using Euclidean distances for absolute gene expression levels and disease indexes, and Z-scores were calculated across rows for normalization. Genes expression levels relative to the *UBC21* gene and to mock-treatment (S13A and S13B, respectively) are shown. Values are means ± SE (n = 3). Experiments were performed three times with similar results.

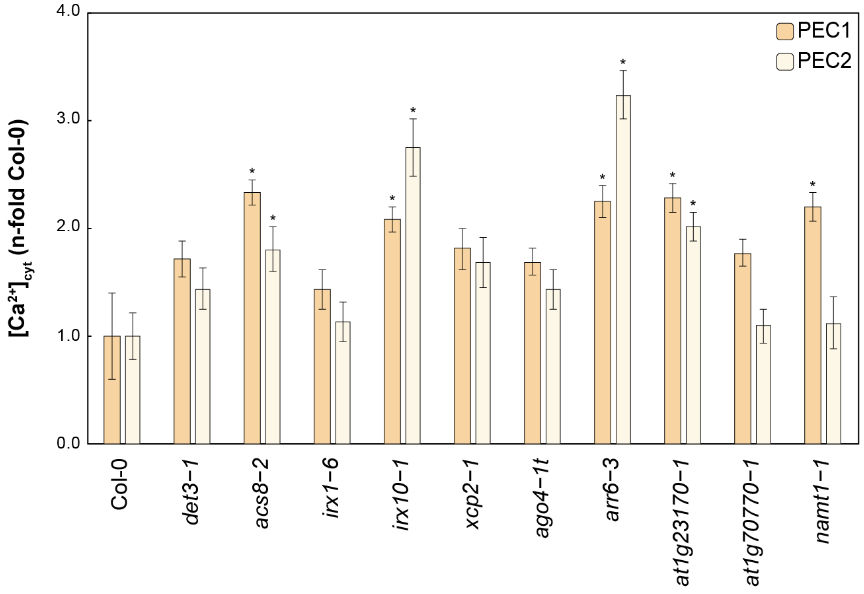

Figure S14. Cell wall derived extracts from pectin fractions (PEC1 and PEC2) of *cwm* plants trigger Ca^{2+} elevations in Col-0 ^{AEQ} plants. Increases in cytoplasmic calcium concentrations ($[Ca^{2+}]_{cyt}$) in seedlings of Col-0^{AEQ} upon treatment with heat treated (121 ºC for 20 min) cell walls extracts (PEC1 and PEC2, 50 ng/µl) from Col-0 wild-type plants and the indicated cell wall mutants. PEC1 corresponds to the weakly bound pectin fraction whereas PEC2 is the highly bound pectin fraction. Data are representative of three independent experiments that gave similar results (means ± SD, n = 16). Asterisks indicate statistically significant differences ($p < 0.05$) with the corresponding fraction from Col-0 plants (ANOVA, Dunnett´s multiple comparisons test correction). Total free Ca^{2+} in PEC1 and PEC2 cell wall fractions used in these analyses was determined by atomic absorption spectrophotometry, but not detectable amounts of Ca^{2+} were found.

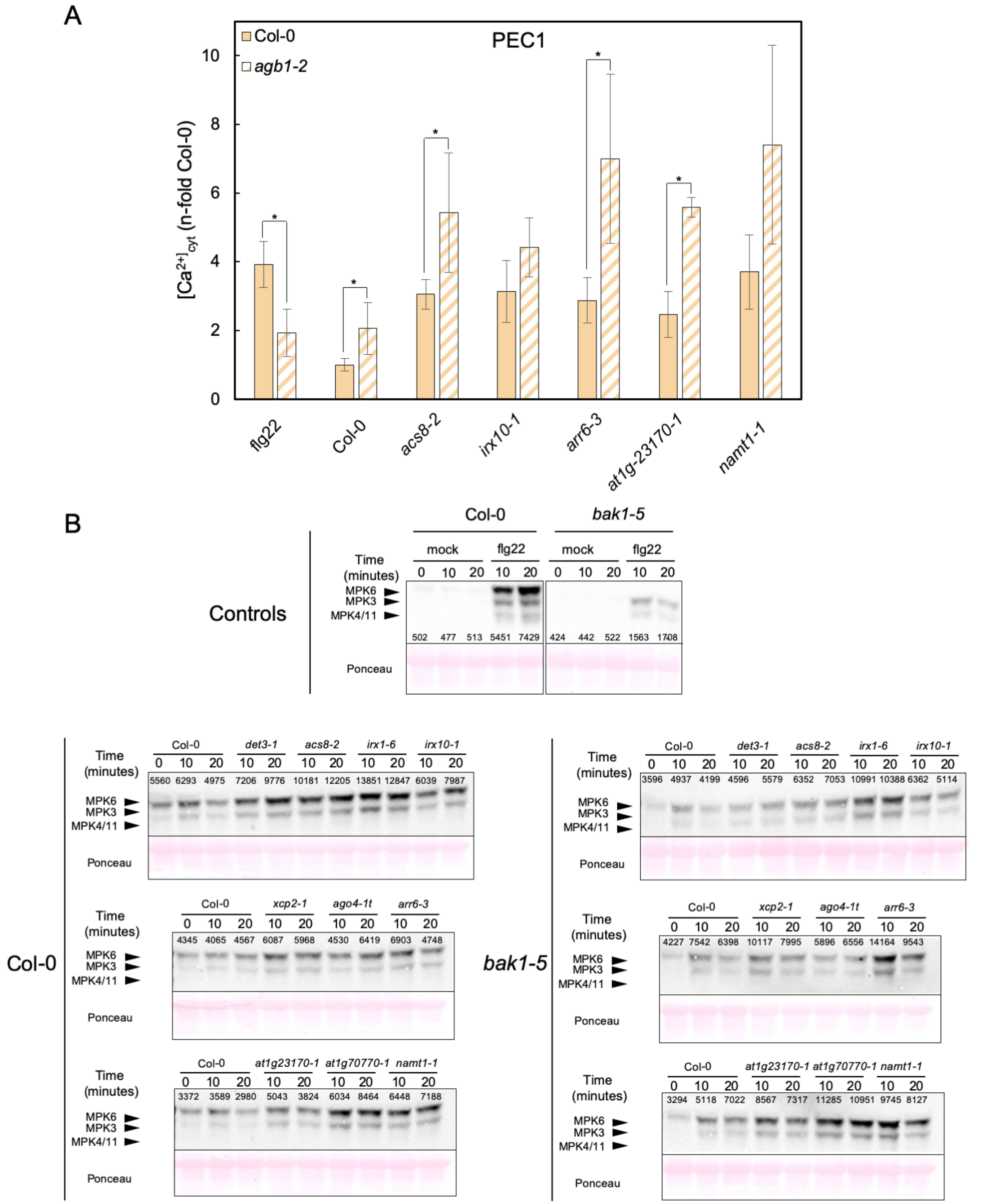

Figure S15. Cell wall derived extracts from PEC1 of cell wall mutant plants trigger immune responses in *agb1-2* and *bak1-5* mutants impaired in the immune regulators AGB1 and BAK1, respectively. (*A*) Increases in cytoplasmic calcium concentrations ($[Ca^{2+}]_{cyt}$) in seedlings of Col-0^{AEQ} and *agb1-2*^{AEQ} lines upon treatment with heat-treated (121 ºC for 20 min) cell wall extracts (50 ng/µl PEC1) from Col-0 and the indicated *cwm* plants (means ± SD, n = 8). Flagellin 22 peptide MAMP (flg22; 1 µM) was used as positive control. Asterisks indicate statistically significant differences between Col-0 and *agb1-2* mutant plants treated with the same fraction or flg22 ($p < 0.1$; ANOVA, Dunnett multiple comparisons test correction). (*B*) Mitogen-activated protein kinases (MAPK) phosphorylation in 12-days old *Arabidopsis* seedlings of Col-0 and *bak1-5* upon treatment with heat-treated (121 ºC for 20 min) cell wall extracts (0.25 ng/µl PEC1) from Col-0 and the indicated *cwm* plants. Western Blot using anti-pTEpY antibody for phosphorylated MAPK moieties at different time points: 0 (seedlings harvested before treatment with PEC1 or flg22), and 10 and 20 minutes after treatment. Arrows indicate the position of MPK6, MPK3 and MPK4/11 proteins. Ponceau red-stained membranes show equal loading. Flg22 (1 µM) and distilled water (mock) were used as controls. Intensity of the bands (relative units) were quantified for each sample with iBright Analysis Software and values are indicated. Additional details of the methodology used for Western analyses are explained in SI Material and Methods.

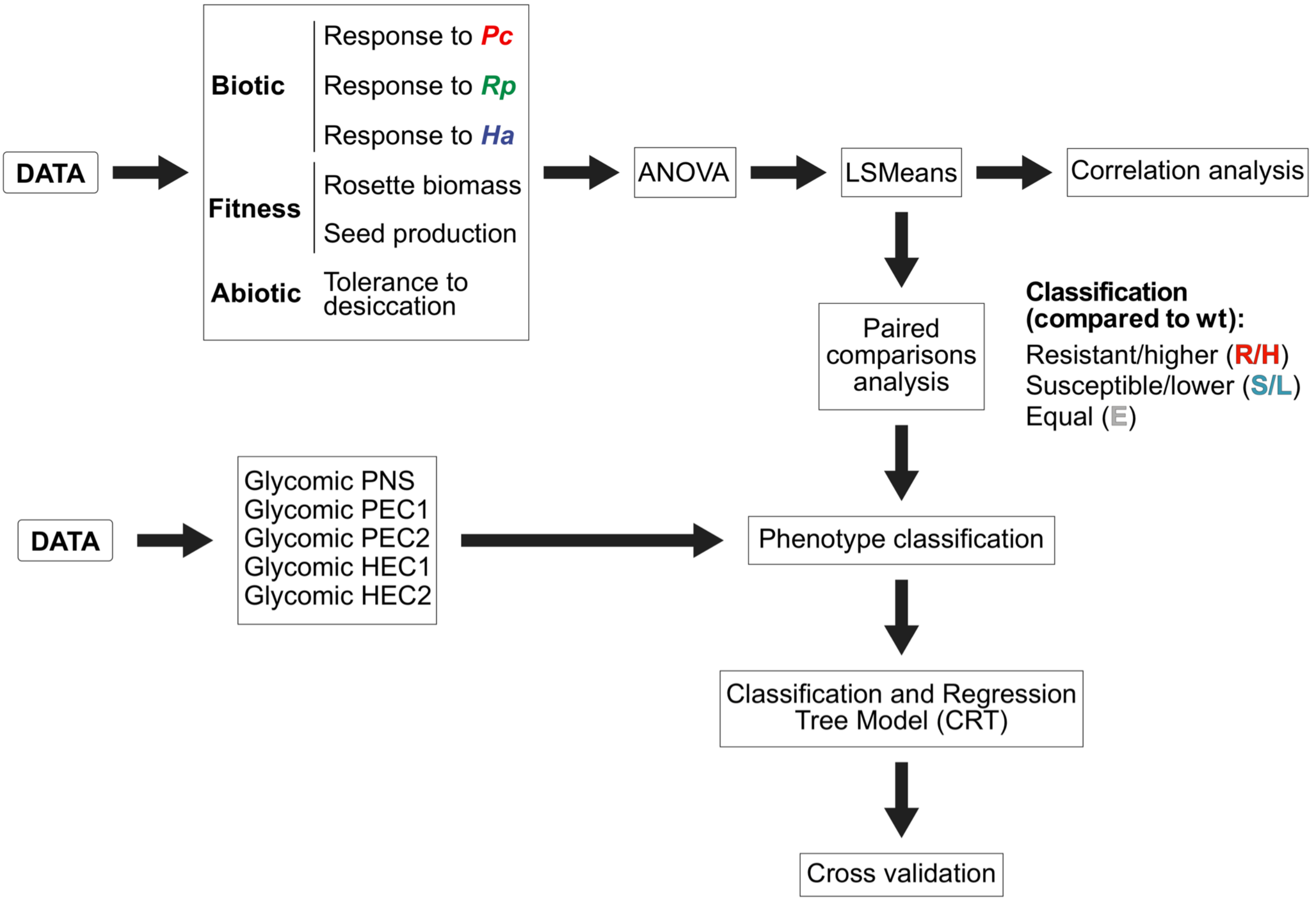

Figure S16. Schema of the mathematical analyses performed to generate the data of Figures 2C, Fig. 4, Fig. S7B, Fig. S10-S12.

Table S1: Summary of the results from the performed Classification and Regression Tree (CRT) analysis. The observed phenotypes and the cell wall fractions used for the analyses are indicated. The monoclonal antibodies which provided a better classification of the observed phenotypes were selected, and the epitope they recognise are shown. In dark green and pale green are indicated the antibodies providing the top 1 and 2 highest CV_ accuracy for the phenotypes analysed, respectvely. Cut-off values are absorbance values that determine the two classification categories. Raw and corrected accuracy values (CV_) are shown. For additional details see SI.

Phenotype	Wall Fraction	Antibody	Recognised wall Epitope	Cut_point	Leaf1_ERS	Leaf2_ERS	OK_E/Total_E	OK_R/Total_R	OK_S/Total_S	Accuracy	CV_Accuracy	CV_AccStd
Pc	PNS	CCRC_M42	Rhamnogalacturonan I	0.1757	11/1/0	5/13/2	11/16	13/14	0/2	75	47.95	6.19
Pc	PEC1	CCRC_M30	Rhamnogalacturonan I	0.0865	0/11/0	16/3/2	16/16	11/14	0/2	84.375	78.53	2.60
Pc	PEC2	CCRC_M106	Fucosylated xyloglucan (Fuc-1,2-alpha-Gal)	0.1393	16/2/0	0/12/2	16/16	12/14	0/2	87.5	84.34	4.18
Pc	HEC1	CCRC_M174	Galactomannan	0.0022	15/2/1	1/12/1	15/16	12/14	0/2	84.375	76.35	4.71
Pc	HEC2	CCRC_M56	Rhamnogalacturonan I	0.6030	13/1/0	3/13/2	13/16	13/14	0/2	81.25	71.07	5.66
Rp	PNS	CCRC_M25	Rhamnogalacturonan I/Arabinogalactan	0.2795	16/1/2	2/9/2	16/18	9/10	0/4	78.125	53.21	4.46
Rp	PEC1	JIM7	Homogalacturonan (GalA1->4MeGalA1->4MeGalA1->4MeGalA1->4MeGalA1->4GalA)	0.8295	17/3/4	1/7/0	17/18	7/10	0/4	75	49.74	5.25
Rp	PEC2	CCRC_M5	Rhamnogalacturonan I	0.2990	0/10/2	18/0/2	18/18	10/10	0/4	87.5	83.88	1.53
Rp	HEC1	CCRC_M114	Xylan	0.0118	0/0/4	18/10/0	18/18	0/10	4/4	68.75	51.97	6.61
Rp	HEC2	CCRC_M69	Rhamnogalacturonan I	0.2380	17/2/4	1/8/0	17/18	8/10	0/4	78.125	50.10	5.42
Ha	PNS	JIM11	Arabinogalactan	0.0543	1/16/0	11/4/0	11/12	16/20	0/0	84.375	61.12	6.06
Ha	PEC1	CCRC_M26	(1,3)(1,6)glucan	0.0030	5/20/0	7/0/0	7/12	20/20	0/0	84.375	72.01	5.45
Ha	PEC2	CCRC_M106	Fucosylated xyloglucan (Fuc-1,2-alpha-Gal)	0.1300	12/4/0	0/16/0	12/12	16/20	0/0	87.5	83.43	2.43
Ha	HEC1	CCRC_M174	Galactomannan	0.0005	7/0/0	5/20/0	7/12	20/20	0/0	84.375	82.96	3.62
Ha	HEC2	CCRC_M138	Xylan	0.2002	4/20/0	8/0/0	8/12	20/20	0/0	87.5	81.34	5.75
Biomass	PNS	CCRC_M22	De-arabinosylated rhamnogalacturonan I	0.4195	13/0/0	3/0/16	13/16	0/0	16/16	90.625	87.31	1.56
Biomass	PEC1	CCRC_M117	Xylan	0.0012	10/0/1	6/0/15	10/16	0/0	15/16	78.125	45.32	5.54
Biomass	PEC2	CCRC_M5	Rhamnogalacturonan I	0.3185	2/0/12	14/0/4	14/16	0/0	12/16	81.25	52.67	4.92
Biomass	HEC1	CCRC_M154	Xylan	0.0075	10/0/0	6/0/16	10/16	0/0	16/16	81.25	64.38	3.89
Biomass	HEC2	CCRC_M69	Rhamnogalacturonan I	0.2072	11/0/1	5/0/15	11/16	0/0	15/16	81.25	54.39	6.05
Seeds	PNS	CCRC_M128	Rhamnogalacturonan I	0.2570	20/0/2	2/2/6	20/22	0/2	6/8	81.25	54.73	6.42
Seeds	PEC1	CCRC_M170	Acetylated mannan	0.0367	20/1/0	2/1/8	20/22	0/2	8/8	87.5	85.07	3.71
Seeds	PEC2	CCRC_M106	Fucosylated xyloglucan (Fuc-1,2-alpha-Gal)	0.1920	22/2/2	0/0/6	22/22	0/2	6/8	87.5	77.53	4.78
Seeds	HEC1	CCRC_M175	Galactomannan	0.0052	21/0/0	1/2/8	21/22	0/2	8/8	90.625	87.62	1.15
Seeds	HEC2	JIM19	Arabinogalactan	0.1258	22/1/2	0/1/6	22/22	0/2	6/8	87.5	62.39	5.07
Drought	PNS	CCRC_M93	Xyloglucan	0.0052	2/6/0	22/2/0	22/24	6/8	0/0	87.5	58.43	6.08
Drought	PEC1	CCRC_M55	Non-fucosylated xyloglucans.	0.0155	17/0/0	7/8/0	17/24	8/8	0/0	78.125	51.74	6.41
Drought	PEC2	CCRC_M5	Rhamnogalacturonan I	0.2990	4/8/0	20/0/0	20/24	8/8	0/0	87.5	65.60	4.61
Drought	HEC1	CCRC_M7	Rhamnogalacturonan I (trimer or larger of beta-(1,6)-Gal carrying one or more Ara residues of unknown linkage)	0.4590	24/5/0	0/3/0	24/24	3/8	0/0	84.375	56.70	5.93
Drought	HEC2	JIM101	Rhamnogalacturonan I	0.0040	24/2/0	0/6/0	24/24	6/8	0/0	93.75	83.16	4.07

Table S2: Oligonucleotides used for T-DNA insertional mutant characterization.

Gene	AGI locus	Background	Allele	Line	Forward oligonucleotide	Reverse oligonucleotide
DET3	*AT1G12840*	Col-0	*det3-2*	SAIL_517_E02	ATCCTCTTGCTCCTCTTCAGC	CTGCGAAATTGAAACCAAAAC
XCP2	*AT1G20850*	Col-0	*xcp2-1*	SALK_057921.45.15.x	GACACTGAGAGGCTGATGAGC	AGCGACCTCTATCGAGTCTCC
			xcp2-2	SALK_010938.56.00.x	AAAGGGAAAAGCTACTGGCTC	GGTTTCCCAGTGTTCCTCTTC
AT1G23170	*AT1G23170*	Col-0	*at1g23170-1*	SALK_046821.54.50.x	TTACGCAAAACCATTGCTACC	GGGAAGATCTAAATGGCGATC
			at1g23170-2	SALK_056368.55.50.x	ACCTTTTGCCTCAAGCTCTTC	ACAGTTTGGATGATGGCTCAG
IRX10	*AT1G27440*	Col-0	*irx10-1*	SALK_055673	ACAAAAGCCGTGATCAATGAC	AAACATCACCAGCACTTCCTG
FRA3	*AT1G65580*	Col-0	*fra3-1*	SAIL_253_E02	TTTGTAAATGAACGTCCCTGC	TACCAACAAATTCCGGTAACG
EXP1	*AT1G69530*	**Ws**	*exp1-1*	FLAG_401A10	GAGGTTGGTGTAACCCTCCTC	TCTCGCTTCGAGAAGGGATAC
AT1G70770	*AT1G70770*	Col-0	*at1g70770-1*	SALK_023673.43.50.x	CAGGAAGTTCCCAAGAGATCC	CCGAATGATGCTCTCACTCTC
			at1g70770-2	SAILK_110286	GTCATCTGTCCGCAAAATGAG	ATCCTTTGGGATTTGGTTTTG
PDF2.1	*AT2G202120*	**Ws**	*pdf2-1-1*	FLAG_441H10	TTCTCTATGCGTTTGATCTCAGC	TTCTGGCATATTTGCAACAAGAAC
AGO4	*AT2G27040*	Col-0	*ago4-1t*	SALK_007523.54.75.x	GAGGTTGGTGTAACCCTCCTC	TCTCGCTTCGAGAAGGGATAC
CTL2	*AT3G16920*	Col-0	*ctl2-1*	SALK_055713.38.85.x	CAGCTTCTTCTCGTCCAACAC	GTTTCGAAACCGCTATTCTCC
SAG21	*AT4G02380*	Col-0	*sag21-1*	SALK_099663.54.70.x	TGGGTCAAAAGACTCAAAGGC	TATGCCAATCAAATTGGAACG
AT4G15160	*AT4G15160*	Col-0	*at4g15160-1*	SALK_007014	TAGTTTCCCAAATTTTTCGGG	GCCCTGGTCAAACAACTAGTG
ACS8	*AT4G37770*	Col-0	*acs8-2*	SALK_066725	ATAACCAACCCATCTAACCCG	GGCTTCTCAACCAGAAAGGTC
			acs8-3	SAIL_102_E05.v1	TCTTGTTCTTGTTCCCATTGG	TCTTCCCAACCCCAAAAATAC
NAMT1	*AT5G04370*	Col-0	*namt1-1*	SALK_001690.56.00.x	GGCCAAGATCATATCCATGTG	TTTTGGTGCGATTTTGGATAC
			namt1-2	SAIL_300_D11	CAATAGCCAGTACCACAACCC	AACAGAGGAACCAAAACCCAC
AT5G51890	*AT5G51890*	Col-0	*at5g51890-1*	SALK_086448.49.80.x	TAGATTCGACACGGTCAAACC	TTTACCTGAATCAAGCCCATG
ARR6	*AT5G62920*	Col-0	*arr6-2*	SALK_008866.55.00.x	TGTTGAGGAAAAATCAGTCGG	CTGCGAGTGAACAGGGTAGAC
			arr6-3	SALK_133123.21.20.x	TCTTCTGGGCCAAATCATATG	TACCGGGCATTGAGTAATCAG
SPT4	*AT5G63670*	**Ws**	*spt4-1*	FLAG_460C02	AAGCGCACCAGCTCAGATTCC	TTCGGCAGAACATATTGTACACGC

Oligonucleotides used for Transgene genotyping and characterization.

APOAEQUORIN	Transgene	Col-0/*agb1-2*	*AEQ*		ATGAAATATGGTGTGGAAACTGATT	GTTGTCTTGTCATCTCATCAACACTC

Table S3: Oligonucleotides used for gene expression analyses (RT-PCR/qRT-PCR)

Gene	AGI locus	Forward oligonucleotide	Reverse oligonucleotide
PHI-1	*AT1G35140*	TTGGTTTAGACGGGATGGTG	ACTCCAGTACAAGCCGATCC
PR1	*AT2G14610*	CGAAAGCTCAAGATAGCCCACA	TTCTGCGTAGCTCCGAGCATAG
PR4	*AT3G04720*	AGCTTCTTGCGGCAAGTGTTT	TGCTACATCCAAATCCAAGCCT
PDF1-2	*AT5G44420*	TTCTCTTTGCTGCTTTCGACG	GCATGCATTACTGTTTCCGCA
LOX3	*AT1G17420*	GCGGAGATTGTTGAAGCGTTT	GCCCCACACCTATTTCTACGGT
PAD3	*AT4G31500*	CAACAACTCCACTCTTGCTCCC	CGACCCATCGCATAAACGTT
LTP3	*AT5G59320*	GAAGAGCATTTCTGGTCTCAAC	GTTGCAGTTAGTGCTCATGGA
PHI1	*AT1G35140*	TTGGTTTAGACGGGATGGTG	ACTCCAGTACAAGCCGATCC
WRKY33	*AT2G38470*	ACGGCCAGAAAGTCGTTAAGG	CATGTCGTGTGATGCTCTCTCC
CYP81F2	*AT5G57220*	TATTGTCCGCATGGTCACAGG	CCACTGTTGTCATTGATGTCCG
AT1G51890	*AT1G51890*	CCAGTTTGTTCTGTAATACTCAGG	CTAGCCGACTTTGGGCTATC
UBQ21	*AT5G25760*	GCTCTTATCAAAGGACCTTCGG	CGAACTTGAGGAGGTTGCAAAG
	AT1G23170	GGGAAGATCTAAATGGCGATC	TTACGCAAAACCATTGCTACC
EXP1	*AT1G69530*	GAGGTTGGTGTAACCCTCCTC	TCTCGCTTCGAGAAGGGATAC
	AT1G70770	AAGTGGCTCTTGCTGGTGC	TTCACCTCCATAACCCCTAAG
AGO4	*AT2G27040*	ATCAGCAAATGGGGCAAACGGG	TTGGACTTTCATTCCCATTGGG
	AT4G15160	AAGGACTCCTCTCTTGGGTTGA	TTCACTGAGTCCTCTTCTGGG
MIEL1	*AT5G51890*	TTCTACTAGATTCGACACGGTC	AACTCTAACTTGTCCAGTCTC
PDF2.1	*AT2G02120*	TTCTCTATGCGTTTGATCTCAGC	TTCTGGCATATTTGCAACAAGAAC
SPT4	*AT5G63670*	AAGCGCACCAGCTCAGATTCC	TTCGGCAGAACATATTGTACACGC

SI References

1. S. Ranf *et al.*, Defense-related calcium signaling mutants uncovered via a quantitative high-throughput screen in Arabidopsis thaliana. *Mol. Plant* **5**, 115–130 (2012).
2. H. Ullah H, *et al.,* The β-subunit of the Arabidopsis G protein negatively regulates auxin-induced cell division and affects multiple developmental processes. *Plant Cell* **15**, 393-409 (2003).
3. S. Sopeña-Torres. Functional characterization of YODA, a mitogen-activated protein kinase kinase kinase (MAP3K) that regulates a novel innate immunity pathway in *Arabidopsis thaliana*. *PhD Thesis*, http://oa.upm.es/37544/ (2015).
4. L. M. Shuman. Fractionation method for soil micronutrients. *Soil Sci*, **140,** 11–22 (1985)
5. S. Pattathil *et al.*, A comprehensive toolkit of plant cell wall glycan-directed monoclonal antibodies. *Plant Physiol*. **153**, 514–525 (2010).
6. Y. Luo *et al.*, V-ATPase activity in the TGN/EE is required for exocytosis and recycling in Arabidopsis. *Nat. Plants* **1**, 15094 (2015).
7. M. Delgado-Cerezo *et al.*, Arabidopsis Heterotrimeric G-protein Regulates Cell Wall Defense and Resistance to Necrotrophic Fungi. *Mol. Plant*, **5**, 98–114 (2012).
8. V. Funk, B. Kositsup, C. Zhao, E. P. Beers, The Arabidopsis xylem peptidase XCP1 is a tracheary element vacuolar protein that may be a papain ortholog. *Plant Physiol*. **128**, 84–94 (2002).
9. U. Avci, H. Earl Petzold, I. O. Ismail, E. P. Beers, C. H. Haigler, Cysteine proteases XCP1 and XCP2 aid micro-autolysis within the intact central vacuole during xylogenesis in Arabidopsis roots. *Plant J*. **56**, 303–315 (2008).
10. B. Zhang *et al.,* PIRIN2 stabilizes cysteine protease XCP2 and increases susceptibility to the vascular pathogen *Ralstonia solanacearum* in Arabidopsis. *Plant J*. **79**, 1009–1019 (2014).
11. J. C. Coates, Armadillo repeat proteins: beyond the animal kingdom. *Trends Cell Biol*. 13, 463–471 (2003).
12. S. Persson, H. Wei, J. Milne, G. P. Page, C. R. Somerville, Identification of genes required for cellulose synthesis by regression analysis of public microarray data sets. *Proc. Natl. Acad. Sci. U.S.A*. **102**, 8633–8638 (2005).
13. D. M. Brown *et al.*, Identification of novel genes in Arabidopsis involved in secondary cell wall formation using expression profiling and reverse genetics. *Plant Cell* **17**, 2281–2295 (2005).
14. Y. Zeng *et al.*, Unique COPII component AtSar1a/AtSec23a pair is required for the distinct function of protein ER export in Arabidopsis thaliana. *Proc. Natl. Acad. Sci*. U.S.A. **112**, 14360–14365 (2015).
15. A. Christensen *et al.,* Functional characterization of Arabidopsis calreticulin1a: A key alleviator of endoplasmic reticulum stress. *Plant Cell Physiol.* **49**, 912–924 (2008).
16. R. Zhong, D. H. Burk, W. H. 3rd Morrison, Z. H. Ye, FRAGILE FIBER3, an Arabidopsis gene encoding a type II inositol polyphosphate 5-phosphatase, is required for secondary wall synthesis and actin organization in fiber cells. *Plant Cell* **16**, 3242–3259 (2004).

17. C. A. Esmon *et al*., A gradient of auxin and auxin-dependent transcription precedes tropic growth responses. *Proc. Natl. Acad. Sci. U.S.A*. **103**, 236–241 (2006).
18. U. G. Schmidt *et al*., Novel tonoplast transporters identified using a proteomic approach with vacuoles isolated from cauliflower buds. *Plant Physiol*. **145**, 216–229 (2007).
19. P. Ranocha, *et al*., Walls are thin 1 (WAT1), an Arabidopsis homolog of *Medicago truncatula* NODULIN21, is a tonoplast-localized protein required for secondary wall formation in fibers. *Plant J.* **63**, 469–483 (2010).
20. N. Denancé *et al.,* Arabidopsis *wat1* (walls are thin 1)-mediated resistance to the bacterial vascular pathogen, *Ralstonia solanacearum*, is accompanied by cross-regulation of salicylic acid and tryptophan metabolism. *Plant J.* **73**, 225–239 (2013).
21. S. Siddique, K. Wieczorek, D. Szakasits, D. P. Kreil, H. Bohlmann, The promoter of a plant defensin gene directs specific expression in nematode-induced syncytia in Arabidopsis roots. *Plant Physiol Biochem*. **49**, 1100–1107 (2011).
22. A. Agorio, P. Vera, ARGONAUTE4 is required for resistance to *Pseudomonas syringae* in Arabidopsis. *Plant Cell* **19**, 3778–3790 (2007).
23. E. Yi Chou, *et al.,* Distribution, mobility, and anchoring of lignin-related oxidative enzymes in Arabidopsis secondary cell walls. *J. Exp. Bot.* **69**, 1849–1859 (2018).
24. C. Montes *et al.,* Cell wall modifications in Arabidopsis thaliana plants with altered □-L-arabinofuranosidase activity. *Plant Physiol.* **147**, 63–77 (2008).
25. C. Sánchez-Rodríguez *et al.,* CHITINASE-LIKE1/POM-POM1 and Its Homolog CTL2 Are Glucan-Interacting Proteins Important for Cellulose Biosynthesis in Arabidopsis. *Plant Cell* **24**, 589–607 (2012).
26. V. B. Busov *et al.,* An auxin-inducible gene from loblolly pine (*Pinus taeda* L.) is differentially expressed in mature and juvenile-phase shoots and encodes a putative transmembrane protein. *Planta*, **218,** 916–927 (2004).
27. J. P. Vogel, T. K. Raab, C. Schiff, S. C. Somerville, PMR6, a pectate lyase-like gene required for powdery mildew susceptibility in Arabidopsis. *Plant Cell* **14**, 2095-2106 (2002).
28. M. Wang, A. Weiberg, E. Dellota, D. Yamane, H. Jin, Botrytis small RNA Bc-siR37 suppresses plant defense genes by cross-kingdom RNAi. *RNA Biol*. **14**, 421–428 (2017).
29. F. M. Salleh et al., A novel function for a redox-related LEA protein (SAG21/AtLEA5) in root development and biotic stress responses. *Plant Cell Environ.* **35**, 418–429 (2012).
30. N. G. Taylor, S. Laurie, S. R. Turner, Multiple Cellulose Synthase Catalytic Subunits Are Required for Cellulose Synthesis in Arabidopsis. *Plant Cell* **12**, 2529–2539 (2000).
31. C. Hernández-Blanco *et al*., Impairment of Cellulose Synthases Required for Arabidopsis Secondary Cell Wall Formation Enhances Disease Resistance. *Plant Cell* **19**, 890–903 (2007).
32. F. Llorente *et al*., ERECTA receptor-like kinase and heterotrimeric G protein from Arabidopsis are required for resistance to the necrotrophic fungus Plectosphaerella cucumerina. *Plant J.* **43**, 165–180 (2005).

33. B. Zhang, H. Liu, X. Ding, J. Qiu, M. Zhang, Z.Chu, Arabidopsis thalianaACS8 plays a crucial role in the early biosynthesis of ethylene elicited by Cu2+ ions. *J. Cell. Sci.* **131**, jcs202424 (2018).
34. R. Wu, F. Zhang, L. Liu, W. Li, E. Pichersky, G. Wang, MeNA, Controlled by Reversible Methylation of Nicotinate, Is an NAD Precursor that Undergoes Long-Distance Transport in Arabidopsis. Mol. Plant. **11**, 1264–1277 (2018).
35. S. R. Turner, C. R. Somerville, Collapsed xylem phenotype of Arabidopsis identifies mutants deficient in cellulose deposition in the secondary cell wall. *Plant Cell* **9**, 689–701 (1997).
36. D. Marino *et al.,* Arabidopsis ubiquitin ligase MIEL1 mediates degradation of the transcription factor MYB30 weakening plant defence. *Nat Commun.* **4**, 1476 (2013).
37. L. M. Fulton, C. S. Cobbett, Two alpha-L-arabinofuranosidase genes in Arabidopsis thaliana are differentially expressed during vegetative growth and flower development. *J. Exp. Bot.* **54**, 2467–2477 (2003).
38. P. M. Szyjanowicz *et al.,* The irregular xylem 2 mutant is an allele of korrigan that affects the secondary cell wall of Arabidopsis thaliana. *Plant J.* **37**, 730–740 (2004).
39. J. López-Cruz et al., Absence of endo-1,4-β-glucanase KOR1 alters the jasmonate-dependent defence response to Pseudomonas syringae in Arabidopsis. *J. Plant Physiol.* **171,** 1524–1532 (2014).
40. Y. Sato *et al.,* Isolation and characterization of a novel peroxidase gene ZPO-C whose expression and function are closely associated with lignification during tracheary element differentiation. *Plant Cell Physiol*. **47**, 493–503 (2006).
41. S. Persson et al., The Arabidopsis irregular xylem8 mutant is deficient in glucuronoxylan and homogalacturonan, which are essential for secondary cell wall integrity. *Plant Cell* **19**, 237–255 (2007).
42. J. P. Vogel, T. K. Raab, C. R. Somerville, S. C. Somerville, Mutations in PMR5 result in powdery mildew resistance and altered cell wall composition. *Plant J*. **40**, 968–978 (2004).
43. D. Chiniquy *et al.,* PMR5, an acetylation protein at the intersection of pectin biosynthesis and defense against fungal pathogens. *Plant J.* **100**, 1022–1035 (2019).
44. R. Slovak *et al.,* Ribosome assembly factor Adenylate Kinase 6 maintains cell proliferation and cell size homeostasis during root growth. *New Phytol.* **225**, 2064–2076 (2020).
45. L. Bacete *et al*., Arabidopsis Response Regulator 6 (ARR6) Modulates Plant Cell-Wall Composition and Disease Resistance. *Mol. Plant Microbe Interact*. **33**, 767–780 (2020).
46. J. Dürr, *et al.,* The transcript elongation factor SPT4/SPT5 is involved in auxin-related gene expression in Arabidopsis. *Nucleic. Acids. Res*. **42**, 4332-4347 (2014).
