# Supplementary material for: Arabidopsis cell wall composition determines disease resistance specificity and fitness
Source: Proc Natl Acad Sci U S A. 2021 Jan 28;118(5):e2010243118. doi: 10.1073/pnas.2010243118 (PMC7865177; doi:10.1073/pnas.2010243118)
Supplement: Supplementary File [file pnas.2010243118.sd02.pdf]

```
# -*- coding: utf-8 -*-
"""
Created on Wed May  6 17:26:55 2020

@author: Tinguaro Rodríguez
"""

"""
Importing/loading libraries. To allow this script running, libraries
numpy, scikit-learn, pandas and graphviz have to be
previously installed.
"""
import numpy as np
from sklearn import tree
import pandas as pd
import graphviz
from sklearn.model_selection import cross_val_score,KFold

"""
Defining the columns of the table (data frame) that will store the
results
"""
result_columns = ['Target', # Target variable
        'Fraction', # Cell-wall fraction of glicomic data
        'Antibody', # Antibody selected by CRT
        'Cut_point', # Cut-point provided by CRT
   'Leaf1_ERS', # Frequency of classes Equal, Resistant and
Susceptible in the first leaf of the tree
   'Leaf2_ERS', # Frequency of classes Equal, Resistant and
Susceptible in the second leaf of the tree
   'OK_E/Total_E', # Rate of correctly classified  observations from
class Equal
   'OK_R/Total_R', # Rate of correctly classified  observations from
class Resistant
   'OK_S/Total_S', # Rate of correctly classified  observations from
class Susceptible
   'Accuracy', # Overall correct classification rate (percentual)
over the training sample
   'CV_Accuracy', # Overall correct classification rate (percentual)
in k-fold cross-validation
   'CV_AccStd'] # Standard deviation of the k accuracy rates
obtained in k-fold cross-validation

"""
Creating the results data frame
"""
results=pd.DataFrame(columns=result_columns)

"""
Cross-validation parameters
"""
k=10 # Number of cross-validation folds
n_reps=100 # Number of replications of k-fold cross-validation
```

```
"""
A parameter to specify whether it is wanted to produce tree graphics
(1) or not (0)
"""
draw_trees=0

"""
Iterables with the names of the data files and fractions to be used,
and target variables to model
"""
filenames=('PNS32.csv','PC132.csv','PC232.csv','HC132.csv','HC232.cs
v')
fractions=('PNS','PC1','PC2','HC1','HC2')
variables=('Pc','Rs','Hpa','Seeds','Biomass','Drought')


"""
Main loops of the fitting and evaluation process:
    First, for each cell-wall fraction, the corresponding glicomic
data is loaded into 'data'
    Column names of the data file are stored, and the glicomic data
itself is placed in 'X' and formatted as float32

    The second loop selects a target variable and stores the
corresponding (categorical) target data in 'y'
    A classification tree (CRT) object is created and fitted to the
X,y data (i.e., for each fraction-target combination)

    The third loop carries out the k-fold cross-validation process,
as many times as defined by the n_reps parameter
    It thus produces a CRT model accuracy estimation at each
replication, each time using a different random partition
        of the data

    Later, back in the second loop, the mean accuracy of the n_rep
cross-validation replications and its std are obtained
    The produced results are printed on the screen, and then
formatted and stored at the 'results' data frame
    A tree graphic is also drawn if specified
"""
for frac in range(0,len(fractions)):
    data = pd.read_csv(filenames[frac],sep=';',decimal=',') # delete
",decimal=','" if working with an Excel setup using '.'
                                                             #
instead of ',' as decimal separator.
    columns=data.columns.get_values()
    feat_names=columns[8:columns.shape[0]]
    X=data.get_values()[:,8:columns.shape[0]] # Explanatory
variables (antibody signals) are placed from the ninth column on.
    X=np.float32(X)

    for var in range(1,len(variables)+1):
        y=data.get_values()[:,var] # The response, classification
variables are placed in columns 2-7
```

```
        tr = tree.DecisionTreeClassifier(max_depth=1,random_state=0)
        treefit = tr.fit(X, y)
        cv_res=np.zeros((n_reps,)) # This vector will store the
cross-validation results of each replication

        for rep in range(0,n_reps):
            kf = KFold(n_splits=k,shuffle=True,random_state=rep) #
Data partition in k folds
            cv = cross_val_score(treefit, X, y, cv=kf) # cv is a
vector with k values (one for each fold)
            cv_res[rep]=cv.mean() # Mean cross-validation accuracy
in this replication

        cv_acc = cv_res.mean() # Mean accuracy of the n_rep
replications
        cv_std = cv_res.std() # Standard deviation of the n_rep
accuracy estimations

        """
        Screen output
        """
        print('*********************************')
        print('Cell-wall fraction: %s' %(fractions[frac]))
        print('Target variable: %s' %(variables[var-1]))
        print('Training accuracy: %s' %(treefit.score(X, y)))
        print('Cross-validation results at each replication: %s' %
(cv_res))
        print('Mean cross-validation accuracy over all replications:
%s' %(cv_acc))
        print('Standard deviation of the accuracy estimations: %s' %
(cv_std))


        """
        Formatting the results
        Three diferent cases are considered since some classes
(Equal, Resistant, Susceptible) do not appear for some target
            variables
        """
        if variables[var-1]=='Hpa' or variables[var-1]=='Drought':
            newline={'Target':'%s' %(variables[var-1]),
                   'Fraction':'%s' %(fractions[frac]),
                   'Antibody':'%s' %
(feat_names[treefit.tree_.feature[0]]), # This recovers the antibody
selected by CRT
                   'Cut_point':treefit.tree_.threshold[0], # This
recovers the cut-point selected by CRT
               'Leaf1_ERS':'%s/%s/0' %
(sum(y[treefit.tree_.apply(X)==1]=='E'),

sum(y[treefit.tree_.apply(X)==1]=='R')),
               'Leaf2_ERS':'%s/%s/0' %
(sum(y[treefit.tree_.apply(X)==2]=='E'),
```

```
sum(y[treefit.tree_.apply(X)==2]=='R')),
               'OK_E/Total_E':'%s/%s' %
(sum(treefit.predict(X[y=='E'])=='E'),sum(y=='E')),
               'OK_R/Total_R':'%s/%s' %
(sum(treefit.predict(X[y=='R'])=='R'),sum(y=='R')),
               'OK_S/Total_S':'0/0', #No Susceptible fenotypes for
Hpa and Drought targets

               #Accuracy is expresed in percentage
               'Accuracy':100*treefit.score(X, y),
               'CV_Accuracy':100*cv_acc,
               'CV_AccStd':100*cv_std}
            class_names=('Equal','Resistant') # Class names for the
tree graphic

        elif variables[var-1]=='Biomass':
            newline={'Target':'%s' %(variables[var-1]),
                   'Fraction':'%s' %(fractions[frac]),
                   'Antibody':'%s' %
(feat_names[treefit.tree_.feature[0]]), # This recovers the antibody
selected by CRT
                   'Cut_point':treefit.tree_.threshold[0], # This
recovers the cut-point selected by CRT
               'Leaf1_ERS':'%s/0/%s' %
(sum(y[treefit.tree_.apply(X)==1]=='E'),

sum(y[treefit.tree_.apply(X)==1]=='S')),
               'Leaf2_ERS':'%s/0/%s' %
(sum(y[treefit.tree_.apply(X)==2]=='E'),

sum(y[treefit.tree_.apply(X)==2]=='S')),
               'OK_E/Total_E':'%s/%s' %
(sum(treefit.predict(X[y=='E'])=='E'),sum(y=='E')),
               'OK_R/Total_R':'0/0', # No resistant fenotypes for
Biomass target
               'OK_S/Total_S':'%s/%s' %
(sum(treefit.predict(X[y=='S'])=='S'),sum(y=='S')),

               #Accuracy is expresed in percentage
               'Accuracy':100*treefit.score(X, y),
               'CV_Accuracy':100*cv_acc,
               'CV_AccStd':100*cv_std}
            class_names=('Equal','Susceptible') # Class names for
the tree graphic

        else:
            newline={'Target':'%s' %(variables[var-1]),
                   'Fraction':'%s' %(fractions[frac]),
                   'Antibody':'%s' %
(feat_names[treefit.tree_.feature[0]]), # This recovers the antibody
selected by CRT
                   'Cut_point':treefit.tree_.threshold[0], # This
recovers the cut-point selected by CRT
               'Leaf1_ERS':'%s/%s/%s' %
```

```
(sum(y[treefit.tree_.apply(X)==1]=='E'),

sum(y[treefit.tree_.apply(X)==1]=='R'),

sum(y[treefit.tree_.apply(X)==1]=='S')),
               'Leaf2_ERS':'%s/%s/%s' %
(sum(y[treefit.tree_.apply(X)==2]=='E'),

sum(y[treefit.tree_.apply(X)==2]=='R'),

sum(y[treefit.tree_.apply(X)==2]=='S')),
               'OK_E/Total_E':'%s/%s' %
(sum(treefit.predict(X[y=='E'])=='E'),sum(y=='E')),
               'OK_R/Total_R':'%s/%s' %
(sum(treefit.predict(X[y=='R'])=='R'),sum(y=='R')),
               'OK_S/Total_S':'%s/%s' %
(sum(treefit.predict(X[y=='S'])=='S'),sum(y=='S')),

               #Accuracy is expresed in percentage
               'Accuracy':100*treefit.score(X, y),
               'CV_Accuracy':100*cv_acc,
               'CV_AccStd':100*cv_std}
            class_names=('Equal','Resistant','Susceptible') # Class
names for the tree graphic

        """
        The replicated cross-validation result for the current
fraction and target is appended to the results data frame
        """
        results=results.append(newline,ignore_index=True)

        """
        Optional graphic of the fitted tree
        """
        if draw_trees==1:
            data_graphic = tree.export_graphviz(treefit,
out_file=None,
                     feature_names=feat_names,
                     class_names=class_names,
                     filled=True, rounded=True,
                     special_characters=True)
            graphic = graphviz.Source(data_graphic)
            treename='%s_%s' %(variables[var-1],fractions[frac])
            graphic.render(treename)


"""
Results are exported to Excel
"""
results.to_excel("results.xlsx")
```
